# Towards Reducing Food Wastage: Analysis of Degradation Products Formed during Meat Spoilage under Different Conditions

**DOI:** 10.3390/foods13172751

**Published:** 2024-08-29

**Authors:** Elisa Uhlig, Matthias Bucher, Mara Strenger, Svenja Kloß, Markus Schmid

**Affiliations:** Sustainable Packaging Institute SPI, Faculty of Life Sciences, Albstadt-Sigmaringen University, Anton-Guenther-Straße 51, 72488 Sigmaringen, Germany; uhlig@hs-albsig.de (E.U.); bucher@hs-albsig.de (M.B.); strenger@hs-albsig.de (M.S.);

**Keywords:** intelligent packaging, meat spoilage, degradation products, food wastage, freshness indicators, food quality and safety

## Abstract

Foodstuffs, particularly perishable ones such as meat, are frequently discarded once the best-before date has been reached, despite the possibility of their continued suitability for human consumption. The implementation of intelligent packaging has the potential to contribute to a reduction in food wastage by enabling the monitoring of meat freshness during storage time independently of the best-before date. The process of meat spoilage is associated with the formation of specific degradation products, some of which can be potentially utilized as spoilage indicators in intelligent packaging. The aim of the review is to identify degradation products whose concentration correlates with meat shelf life and to evaluate their potential use as spoilage indicators in intelligent packaging. To this end, a comprehensive literature research was conducted to identify the factors influencing meat spoilage and the eight key degradation products (carboxylic acids, biogenic amines, total volatile basic nitrogen, aldehydes, alcohols, ketones, sulfur compounds, and esters) associated with this process. These degradation products were analyzed for their correlation with meat shelf life at different temperatures, atmospheres, and meat types and for their applicability in intelligent packaging. The review provides an overview of these degradation products, comparing their potential to indicate spoilage across different meat types and storage conditions. The findings suggest that while no single degradation product universally indicates spoilage across all meat types and conditions, compounds like carboxylic acids, biogenic amines, and volatile basic nitrogen warrant further investigation. The review elucidates the intricacies inherent in identifying a singular spoilage indicator but underscores the potential of combining specific degradation products to expand the scope of applications in intelligent packaging. Further research (e.g., storage tests in which the concentrations of these substances are specifically examined or research on which indicator substance responds to these degradation products) is recommended to explore these combinations with a view to broadening their applicability.

## 1. Introduction

Meat is a highly perishable foodstuff. This is due to its high nutrient content (e.g., lipids and proteins), which favors microbial growth, the high amount of available water (a_w_ > 0.85), a pH value of >4.6 and the presence of autolytic enzymes [1]. Additionally, meat is a heterogeneous product, exhibiting a diverse range of structural characteristics even among individuals of the same species. The variation observed within a single animal is attributable to the differing compositions of connective tissue, proteins, and adipose deposits within specific muscle groups [2]. Furthermore, there are differences in the composition of meat between different animal species [3]. In the case that the referenced literature contains the requisite information, this review differentiates between various animal species (e.g., beef and poultry), which can also be classified as red (e.g., beef) or white (e.g., poultry) meat based on their distinct compositional characteristics. The color of meat results from the different concentrations of myoglobin present in different types of meat [4]. Meat analogs, which are processed foods that imitate meat products [5], and meat products are excluded from this review.

The process of meat spoilage is a complex process that renders meat unfit for human consumption. Meat spoilage is caused by physical changes during processing, chemical and biological agents (e.g., lipid oxidation, enzymatic reactions by lipases and proteases) and/or microbial activity [6,7]. The process of meat spoilage results in the formation of degradation products. It is essential to provide a clear definition of the term ‘degradation products’ in this review, as the term is used inconsistently in the literature. Degradation products are defined as chemical substances formed during the chemical degradation or partial degradation of substances [8]. The pH value, the substantial quantity of free water present (a_w_ value) and the high nutrient content of meat facilitate the growth of a diverse range of microorganisms. The growth of microorganisms is influenced by a number of factors, including the storage conditions. The most significant of these is the atmospheric conditions, especially the amount of free oxygen and carbon dioxide, and the temperature [9,10]. Moreover, the initial microbial load, particularly of bacteria, and the slaughtering process are of significant consequence with regard to the microbiological quality of meat [11]. The definition of the point at which meat is considered to have spoiled is dependent on two key aspects: the sensory and microbiological conditions of the meat. If the colony forming unit per square centimeter (CFU/cm^2^) value rises above 5 × 10^6^ to 10^8^, the meat is officially considered spoiled [12]. In numerous countries, including those within the European Union, there are legally defined limits for various microorganisms in carcasses of different meat types set forth in Regulation (EC) 2073/2005 on microbiological criteria for foodstuffs. For instance, the regulation specifies a limit of 2 CFU/cm^2^ of *Enterobacteriaceae* for pork carcasses [13]. It is essential to consider the specific limits of microbial species, as some microorganisms, such as lactic acid bacteria (LAB), can also act as protective agents. Once the shelf life has been exceeded, the product may exhibit sensory impressions such as ‘buttery’, ‘sour’ and ‘greasy’ characteristics. The initial sensory indications of spoilage in beef (at 10 °C) are primarily discernible through the perception of the aforementioned characteristics, whereas in chicken (at 4 °C), the orthonasal impression of freshness serves as the primary indicator [14]. In general, changes in color, texture, the formation of liquid or slime and off-flavors are described as signs of meat spoilage [15]. The ‘best before’ date and the ‘use by’ date can be determined according to ISO 16779:2015-08 Sensory analysis—Assessment (determination and verification) of the shelf life of foodstuffs [16]. An alternative approach to determining the expiry date is the ‘quality index method’, which also assesses non-microbial spoilage by evaluating a range of quality parameters that change during storage [17].

In an empirical study, consumers consistently indicate that one of the primary reasons for discarding food items is that they have exceeded their best before date. It is not uncommon for consumers to lack confidence in the safety of foodstuffs [18]. Food packaging affects the shelf life and therefore the quality and safety of food. Inappropriate packaging is a significant contributor to food wastage, which has a range of adverse environmental impacts and social implications, particularly as the global population continues to grow and the need for safe and secure food supplies increases. Conversely, optimized packaging contributes to environmental, economic and social sustainability and is therefore an essential element in any sustainable development strategy [19].

The concept of smart packaging, especially in the context of food, has gained considerable traction since 2017. The advent of the COVID-19 pandemic served to further accelerate the pace of research and development in this field [20]. The term ‘smart packaging’ is used to describe packaging that is equipped with the ability to monitor and assess the food conditions it contains as well as the environment within the packaging [21], thereby ensuring higher product safety and quality standards. Moreover, the implementation of smart packaging has the potential to enhance sustainability throughout the entirety of product’s value chain [22], as it may contribute to a reduction in food wastage. Two types of smart packaging exist: active and intelligent packaging [20]. Active packaging is packaging that absorbs or releases substances to extend the product’s shelf-life [23]. For example, the packaging is designed to gradually release substances to the food, thereby increasing its resistance to oxygen [24]. In contrast, intelligent packaging is used to monitor food or its environment [23] during storage and/or transport of the product [25]. The implementation of intelligent packaging concepts has the potential to enhance food safety and reduce avoidable food wastage [26,27]. As previously stated in the introduction, in addition to the term ‘degradation products’, there is also a definitional gap for the terms ‘indicator’ and ‘indicator substance’. The aforementioned gap is subsequently addressed in this paper. In the context of smart packaging, an indicator is defined as a device that provides information about food quality, microbial activity and/or other characteristics. Indicators are designed to respond to the presence, absence or changing concentrations of chemical and/or biological substances. The response of the indicator is often discernible through a change in color [8]. Consequently, in this review, the term ‘indicator substance’ is defined as a substance that reacts to the presence and the change in concentration of degradation products formed during the change (spoilage processes) of packaged products. In order for an indicator to be used for the purpose of indicating meat spoilage, it must meet a number of specific requirements. To guarantee quality and safety, the utilization of methods for testing the freshness of meat offers a viable option, provided that they are cost-effective, non-destructive, efficient [28] and precise [29]. It is further required that indicator substances and degradation products do not react too slowly, but rather indicate meat spoilage at the earliest possible juncture. Moreover, the indication spectrum must be as extensive as possible [30], with a sufficiently large number of degradation products to ensure the ability to make an accurate diagnosis [31]. It is possible for indicators to be in direct contact with the product, as well as in the gas phase of the headspace of the packaging [32]. In either case, the indicators must be capable of being integrated into the packaging material and must not present any harmful effects [29]. When employing indicators for consumer applications, it is of paramount importance that the resulting indicator reactions are readily comprehensible by the consumer [29,33]. Therefore, it is essential to identify suitable degradation products that elicit these reactions in order to monitor meat spoilage.

It is possible to predict which microorganisms will grow or dominate meat spoilage during storage if certain chemical and physical parameters are known. Moreover, the range of degradation products generated by these microorganisms, encompassing esters, ketones, aldehydes, sulfur compounds, amines, and volatile fatty acids, can be anticipated [11]. This knowledge is essential for the identification of appropriate indicators for intelligent packaging concepts. The aim of this review is to present an overview of the search for suitable degradation products whose concentration correlates with the shelf life of meat, and to evaluate their potential use as indicators of meat spoilage. To this end, a comprehensive literature review is carried out and the aforementioned parameters (temperature, atmosphere, meat type) and degradation products (carboxylic acids, biogenic amines, total volatile basic nitrogen, aldehydes, alcohols, ketones, sulfur compounds, and esters) are determined in order to ascertain suitable degradation products for indicating meat spoilage (hereinafter referred to as ‘degradation products’)packaging concept.

In light of the review, the following steps may then be undertaken: A systematic analysis should be conducted to determine the extent to which intelligent packaging can enhance the shelf life and safety of meat. This will entail identifying specific degradation products that are indicative of meat spoilage. It is recommended that the potential integration of these degradation products into intelligent packaging concepts be evaluated by investigating the influence of different factors, such as temperature, atmosphere and meat type. In addition, a clear understanding of the mechanisms underlying meat spoilage and the effectiveness of different degradation products as indicators for intelligent packaging applications should be gained. The development of more accurate and consumer-friendly spoilage detection methods will contribute to reducing food wastage and improving food safety and packaging concept.

## 2. Materials and Methods

The initial search was conducted using Google Scholar, which yielded a comprehensive range of scientific studies on the microbiological degradation products of meat and their potential role in shelf life indication systems. In conclusion, studies published between 1987 and 2022 were cited. In the case of basic literature, older literature up to 1988 was cited in individual cases where no more recent literature could be found.

Firstly, the factors influencing meat spoilage were identified and defined. The principle search terms employed for this purpose were ‘intrinsic factors’ ‘extrinsic factors’ ‘processing factors’ ‘implicit factors’ and ‘emergent factors’. Secondly, the degradation products of meat spoilage (aldehydes, alcohols, ketones, sulfur compounds, esters, carboxylic acids, biogenic amines, total volatile basic nitrogen) were identified and their influence on the factors ‘temperature’, ‘atmosphere’ and ‘meat type’ was investigated. In this review, the most commonly accepted trivial names are used to refer to chemical compounds and the International Union of Pure and Applied Chemistry (IUPAC) name is provided in brackets when used for the first time, unless it is identical to the commonly accepted trivial name. The primary focus has been on the collation and evaluation of scientific literature on these degradation products in the context of meat spoilage and their potential integration into intelligent packaging concepts, particularly for consumer use packaging concept. In addition to the names of degradation products, the primary search terms included ‘meat’, ‘spoilage’, ‘microbial’, ’microorganism’ ‘intelligent packaging’, ‘smart packaging’, ‘shelf life’, ‘vacuum’, ‘modified atmosphere’, ‘air’, ‘biogenic amines index’, ‘Total Volatile Basic Nitrogen (TVB)-N spoilage limits’, which were searched individually and in combination with one another. Finally, the results are discussed in order to reach a conclusion and outlook for future research.

## 3. Basics of Microbial Spoilage in Meat—Influencing Factors

**Figure 1:**
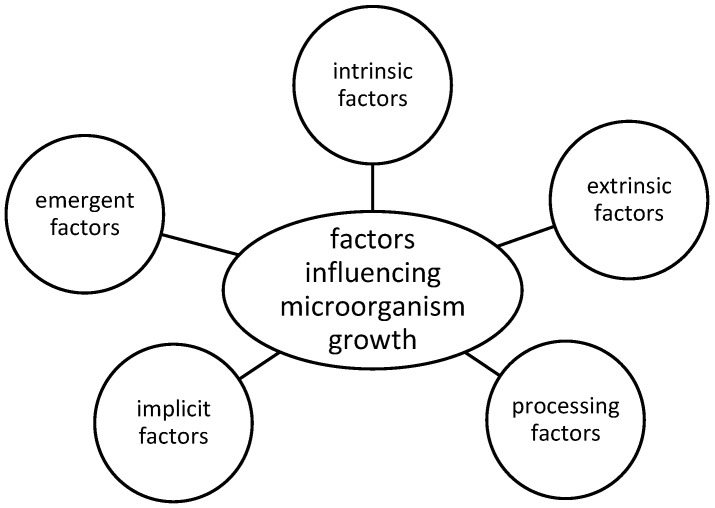
Overview of different factors (intrinsic, extrinsic, processing, implicit, emergent) influencing meat spoilage by affecting microorganism growth.

The contamination of meat with microorganisms, such as bacteria is a natural consequence of a number of factors, including animal diseases, the slaughtering process and the opening of the carcass. These microorganisms can originate from a range of sources, including cutting knives, lymph nodes and bacteria present in the intestines [1]. The types of bacteria that grow during meat processing are contingent upon the storage conditions of the meat in question [33]. The growth of microorganisms is influenced by a multitude of factors, as illustrated in Figure 1 and can be classified into the following categories: intrinsic, extrinsic, processing, implicit and emergent factors. The intrinsic factors are related to the physicochemical properties and structure of meat [34]. To illustrate, the quantity of glycogen has an impact on the rate of spoilage of meat [2]. The extrinsic factors include all factors that are influenced by the type of packaging and storage conditions, such as temperature and/or atmosphere [35]. The processing of the product exerts an influence on the primary microbial community [36]. Implicit factors are intrinsic biotic parameters, such as the synergistic or antagonistic interactions between microorganisms [37]. The last category of emergent factors comprises those that exert a greater influence when interacting with other factors than when acting in isolation [34].

Meat spoilage is dependent on a microbiota which is dominated by psychrophilic microorganisms [38]. Under aerobic conditions, the predominant microorganisms associated with spoilage of red and white meat are *Pseudomonas* spp. [39,40,41,42,43,44,45]. Under anaerobic conditions lactic acid bacteria (LAB), such as *Lactobacillus*, are the primary agents responsible for the spoilage of meat [46]. These LAB represent a dominant population in anaerobic packaging [1,47,48]. In addition to LAB, anaerobic *Brochothrix* spp. are present in modified atmosphere packaging (MAP) with a low oxygen content and vacuum packaging [39,49]. A key distinction between LAB and *Brochothrix* spp. is the threshold at which meat spoils. LAB will cause spoilage at a concentration of 10^8^ CFU/g, whereas *Brochothrix* spp. are capable of causing spoilage at significantly lower germ counts [6]. The presence of facultative anaerobic *Enterobacteriaceae* is indicative of the freshness of the meat and reflects the hygienic conditions under which the meat was produced and transported. *Enterobacteriaceae* is capable of growing under both aerobic and anaerobic conditions. However, two studies have reported that the growth of *Enterobacteriaceae* is more pronounced in modified atmosphere packaging than in vacuum-packed meat [39,50]. This is particularly evident in pork stored at 5 °C [39]. Another bacterium associated with meat spoilage is *Clostridium* spp. Some strains are capable of surviving under psychrophilic conditions. *Clostridium* spp. has been identified as a potential cause of blown vacuum packaging and it is a spore-forming bacterium [51,52]. 

Molds and yeasts are infrequently implicated in meat spoilage, and are thus receive minimal attention in this review. The species isolated from fresh and spoiled meat include *Candida*, *Cryptococcus*, *Debaryomyces* and *Yarrowia*. *Trichosporon* spp. is exclusively associated with spoiled poultry [33].

In addition to the main spoilage organisms previously described, there are several other bacterial genera that have been identified, including *Acinetobacter* spp., *Psychrobacter* spp. and *Moraxella* spp. The majority of these organisms demonstrate optimal growth under aerobic conditions and exhibit a tendency to increase in proportion at ambient temperatures [39]. Table 1 provides an overview of a selection of the microorganisms that are responsible for meat spoilage under aerobic, anaerobic and facultative anaerobic conditions.

### 3.1. Extrinsic Factors

Temperature is a factor that affects the shelf life of meat [55]. The utilization of intelligent packaging represents a potential solution for providing consumers with reliable food safety information, particularly given that the temperature within domestic refrigerators frequently exceeds the optimal maximum temperature for meat storage, which is 4 °C [53,54]. This is corroborated by a study on domestic refrigerator storage temperatures, which revealed that 71% of participants had a refrigerator temperature higher than 5 °C [54]. Temperature has a direct effect on microbial growth and the species of microorganisms present [56,57]. Accordingly, temperature has a more pronounced influence on the total viable count (TVC) of microorganisms compared to the composition of the headspace within the packaging [58,59]. It is therefore crucial to ensure the maintenance of the cold chain throughout the entire fresh meat supply chain [57] in order to effectively inhibit the growth of pathogens and prevent the spoilage of the meat [60]. Consequently, the maximum temperatures permitted for transportation and storage of goods, including meat, are regulated in a number of countries. In Europe, for example, the core temperature of meat must not exceed 7 °C during the transportation and storage [61,62]. In the USA, the maximum temperature permitted during the transportation and storage of meat is 5 °C [60].

The storage atmosphere within the packaging is an important factor in extending the shelf life of fresh meat [63,64]. For example, in MAP, the atmosphere surrounding the product has been replaced by oxygen (O_2_), carbon dioxide (CO_2_) and/or nitrogen (N_2_) [65,66]. The microbial growth of microorganisms within the package can be influenced by the presence of different gases, primarily oxygen (O_2_) and carbon dioxide (CO_2_), within the headspace. CO_2_ has been demonstrated to possess antimicrobial properties, especially against gram-negative aerobes such as *Pseudomonas* spp. [65]. Consequently, the utilization of CO_2_ in MAP retards the spoilage of meat [67] by curbing the growth rate [39,68]. On the one hand, reducing the level of O_2_ to a minimum can enhance the shelf life of meat in MAP [69] and maintain the red color of fresh red meat [19]. On the other hand, high levels of O_2_ (up to 70%) also help inhibit the growth of strict anaerobic bacteria like *Clostridium* spp. Nevertheless, numerous prevalent food spoilers have been observed to exhibit resistance to these atmospheres [70]. In addition, a high O_2_ content in the packaging atmosphere is deleterious to sensory properties, as it may induce lipid oxidation, resulting in the development of rancid off-flavors [65]. In addition to the effects of O_2_ and CO_2_ on microorganisms described above, effects can be indicated even in the absence of O_2_ and CO_2_. These effects mainly occur in vacuum-packed meat and in MAP with 100% N_2_ as shown by [71], who conducted a comparative analysis of various studies on meat packaging atmospheres and concluded that vacuum-packed and meat packed with 100% N_2_ (a rarely employed experimental approach) exhibit a delayed spoilage compared to meat packed with O_2_ and CO_2_. Nevertheless, N_2_ has a negligible impact on microbial growth. largely due to its extremely low solubility in both fat and water [72]. The absence of O_2_ in such packaging concepts mainly affects aerobic bacterial species such as *Pseudomonas* spp., *Acinetobacter* spp. and *Psychrobacter* spp. [67,70]. However, the growth of facultative anaerobic food spoilage organisms, including LAB [71] and anaerobic ones such as *Clostridium* spp. [51] remains unaffected.

### 3.2. Processing Factors

The processes during the meat processing, from the breeding of animals to the final packaging of meat, are of great importance with regard to the composition of the meat as well as the TVC of microorganisms present. The contamination of meat is related to the environmental conditions under which the meat is handled and processed [36]. One contributing factor is the ability of bacteria to adhere to surfaces and form biofilms, which can serve as a source of contamination during processing [73]. Surface characteristics exert a decisive influence on the adhesion of bacteria and the subsequent formation of biofilms [74,75]. Other process-related factors that exert an influence on the rate of meat spoilage include the temperature during deboning and carcass cutting [76]. The influence of process factors is also reflected in the different microbial compositions across different slaughterhouses [3].

### 3.3. Intrinsic Factors

The intrinsic factors that contribute to meat spoilage are those that pertain to the physicochemical properties of the meat. The growth of microorganisms on meat is influenced by parameters such as pH value, water activity [77], available nutrients, and antibacterial elements [78], e.g., antibiotics. The growth of microorganisms has been observed to vary in response to different pieces of meat from different parts of the animal [39] as well as between different animal species [3]. These differences in meat spoilage are mainly due to differences in glycogen reserves and the accessibility of micronutrients and minerals, including iron [2].

### 3.4. Implicit Factors

The implicit factors are those that are themselves related to microorganisms [79]. The most intensively studied group of bacteria are LAB, which are capable of producing a range of inhibitory substances, including bacteriocins [80,81]. In addition, metabiotic, commensalism, predation and competition of microbes are among implicit factors. The term ‘metabiotic association’ is used to describe the phenomenon whereby presence of one microorganism creates an environment conducive to the growth of another microorganism. The term ‘commensalism’ is used to describe the interaction of two groups: one that benefits and one that is not affected. In contrast, the term ‘predation’ is used to describe an organism that feeds on other organisms [79].

In addition to the aforementioned factors, there are also emergent factors that warrant consideration. To date, there has been a paucity of research conducted on these factors. One study offers a definition what is meant by ‘emergent factors’: When factors have a greater effect when interacting than when acting alone, they are designated as emergent factors [34].

## 4. Degradation Products That Can Be Indicated during Meat Spoilage

The spoilage of meat is regularly accompanied by the formation of a range of degradation products, which are the result of the underlying changes that occur during the spoilage process [30]. The aforementioned degradation products can be utilized to indicate the process of spoilage, for example, in the context of food analysis, shelf-life modeling or intelligent packaging concepts. A variety of spoilage indicators can be indicated, during the analysis of spoiled meat [14]. The following categories have been identified for the classification of typical spoilage products: Biogenic Amines (BAs), Total Volatile Basic Nitrogen (TVB-N) and Volatile Organic Compounds (VOCs) encompass a range of compounds, include aldehydes, alcohols, ketones, esters, sulfur compounds and carboxylic acids.

Several studies show a correlation between the release of VOCs associated with spoilage and the growth of specific microbial species during the storage of meat [82,83]. Furthermore, these studies demonstrate that the generation of VOC is dependent on the on strain type involved [83,84]. The external factors influencing meat spoilage, such as the packaging atmosphere, have a significant impact on the profile of VOCs formed [85,86]. This phenomenon can be attributed to the impact of the atmosphere on the microorganisms that grow in it. However, it is also influenced by to other processes such as the accelerated oxidation of lipids at higher O_2_ concentrations [87]. In addition, a correlation is identified between the VOC profile and the fatty acid composition of the meat [88].

The spoilage characteristics become observable when the microorganisms have metabolized the glucose ((2R,3S,4R,5R)-2,3,4,5,6-Pentahydroxyhexanaldehyde) and lactate (2-hydroxypropanoate) present in the meat and the microorganisms begin to utilize the proteins [56]. The initial indicators of meat spoilage are the presence of decomposition products, such as sulfides, methyl esters or ammonia (azane). These compounds result from the conversion of amino acids [6] and lead to sensory signs of spoilage like off-flavors [89].

### 4.1. Aldehydes

Aldehydes are formed through a number of different processes, including triglyceride hydrolysis, oxidation of unsaturated fatty acids, and lipid autooxidation in meat. These processes can occur under a variety of conditions, including air, vacuum, or modified MAP. Hexanaldehyde (hexanal), nonanaldehyde (nonanal), heptanaldehyde (heptanal), benzaldehyde, and Isovaleraldehyde (3-methylbutanal) are the most commonly occurring aldehydes in naturally spoiled meat and in inoculated model meat systems [11]. The formation of aldehydes in meat during storage and spoilage is the result of lipid oxidation and/or processes catalyzed by growing bacteria [90,91], which is influenced by the amount of O_2_ in the packaging atmosphere [92,93,94]. The highest amounts of aldehydes are produced by *Pseudomonas* spp., *Carnobacterium* spp. and *Enterobacteriaceae* [11,90]. However, not all commonly found aldehydes are linked to bacterial growth. Otherwise, the low absolute concentration of aldehydes and their rapid oxidation to acids at an early stage of storage would be inexplicable [11]. Consequently, the concentration of aldehydes such as hexanaldehyde is not a reliable indicator of meat spoilage since this phenomenon cannot be solely explained by microbiological influences. Moreover, aldehydes are found in both sterile and spoiled pork [95]. A comparable estimation is conducted for chicken fillets in different packaging atmospheres. Although a correlation cannot be established between aldehyde concentration and meat storage time due to the high degree of variability in the results [90].

#### 4.1.1. Temperature

The existing literature indicates that the concentration of aldehydes during the storage of meat increases with rising temperature, regardless of the storage atmosphere [15,82]. Nevertheless, the impact of temperature on aldehyde formation is seldom addressed in the existing scientific literature.

#### 4.1.2. Atmosphere

The two aldehydes acetaldehyde and 2-butenal are not yet widely used as indicator substances, due to the limitations imposed by the packaging atmosphere and the composition of the growing microorganisms, which are influenced by it. The results indicate, that 2-butenal ((2E)-but-2-enal) is a more suitable degradation product for meat packed aerobically, while acetaldehyde is more appropriate for vacuum-packed meat [95]. This limits the applicability of aldehydes as a universal degradation product for diverse atmospheric conditions and for a range of products. A comparable dependence of aldehydes on packaging atmospheres and their O_2_ contents is observed. Malonaldehyde (propandial) in beef, for example, demonstrates a continuous increase exclusively under aerobic conditions [36]. Aldehydes are also indicated when beef is stored at 4 °C under vacuum. This correlates with the increase in *Lactobacillus* [46]. Another study of beef storage at 4 °C under MAP (70% O_2_, 20% CO_2_, 10% N_2_) found a significant increase in aldehydes and recommended octanal, nonanal, 2 and 3-methylbutanal as degradation products of beef spoilage [86]. When studying the evolution of 3-methylbutanal during the storage of poultry in MAP (40% CO_2_, 30% N_2_, 30% O_2_) at 4 °C, a significant increase can be indicated during the first days. Therefore, 3-methylbutanal can be used for the early indication of spoilage in chickens packed in MAP [96]. The selection of aldehydes as indicators is restricted to the extent of fat oxidation, as exemplified by malondialdehydes [97]. However, this approach cannot be extrapolated to microbial conditions without further research. Various aldehydes display a dependence on specific packaging atmospheres and their oxygen levels. Consequently, it is challenging to identify a universally applicable aldehyde for indicating meat spoilage [92].

#### 4.1.3. Meat Type

A trend analysis of acetaldehyde and 2-butenal in air- and vacuum-packed meat (beef, pork, and poultry) at 4 °C indicates that these two aldehydes indicates an increase over the storage time in all meat types. The formation of these compounds is more prominent in beef during the first days of storage, whereas the subsequent increase is less prominent in pork and poultry [98]. Hexanaldehyde is the dominant aldehyde in chicken meat [33], showing a continuous increase in concentration during the seven days storage period of chicken meat [99]. The differences in the profile of aldehydes formed can be attributed to the varying composition of fatty acids available for oxidation, which depends on the type of meat. Hexanal, for instance, is the result of linoleic acid oxidation [88]. A review of the literature reveals a paucity of information regarding the formation of aldehydes and the spoilage of different types of meat.

### 4.2. Alcohols

Microbial metabolism plays a crucial role in facilitating the breakdown of proteins and amino acids, as well as the reduction of ketones and aldehydes derived from lipid peroxidation. This process ultimately leads to the formation of alcohols [1]. An increase in microbial counts has been observed to result in a greater increase in alcohols in stored meat [90]. As an example, a significant increase in ethanol and 2-propanol (propan-2-ol) is indicated in chicken meat after 12 days at 4 °C [96]. The evolution of ethanol and methanol in beef and poultry under a modified atmosphere (70% O_2_ and 30% CO_2_) is also observed, and a slight increase is indicated during the first seven days. The formation of alcohols increases marginally with extended storage periods, yet the overall level remains notably low. In beef, a notable increase in ethanol levels is only observed at elevated storage temperatures of 10 °C and longer storage periods [14]. A concentration increase over time would be advantageous as it would allow for a correlation between alcohol content and microbial spoilage. Given that ethanol formation is contingent upon the presence of specific microorganisms [100], it is plausible that this phenomenon may also be associated with the microbial flora. The main microorganisms responsible for alcohol production are *Pseudomonas* spp. and *Carnobacterium* spp. [1]. In chicken, 3-methyl-1-butanol (3-Methylbutan-1-ol) correlates more strongly with the growth of several spoilage bacteria, e.g., *Pseudomonas* spp., than ethanol [101]. The Pearson correlation coefficients, which quantify the strength of the linear correlation between two variables [102], show relatively weak correlations between ethanol and the total viable count (TVC) (r = 0.66) and between ethanol and psychrotrophic microorganisms or *Pseudomonas* spp (0.6) [101]. Different bacterial strains can even produce different profiles of alcohols [83]. For example, not all strains of *Carnobacterium maltaromaticum* are capable of forming alcohols under aerobic (air) and/or anaerobic (vacuum) conditions. The alcohols 1-octen-3-ol and 2-ethyl-1-hexanol (2-ethylhexan-1-ol) are produced by the greatest number of strains, irrespective of the applied atmosphere. The number of observed strains for 3-methyl-1-butanol is consistent, but only under anaerobic conditions [103].

However, other alcohols such as 1-octen-3-ol (oct-1-en-3-ol) and 1-pentanol (pentan-1-ol) are also formed in the absence of microorganisms in sterilized pork [87]. As a result, these alcohols are not suitable as degradation products for the spoilage process. It is therefore not always reliable to draw conclusions about microbial spoilage levels based on these alcohols.

#### 4.2.1. Temperature

The formation of various VOCs, including alcohols, in poultry under MAP (70% O_2_ + 30% CO_2_) is influenced by temperature. When it is stored at 4 °C, a correlation between spoilage and ethanol formation is evident. However, at 10 °C the presence of ethanol cannot be confirmed. The concentrations measured at 4 °C are already very close to the limit of quantification in the range around the critical limit, and the confidence intervals chosen for the individual measuring points are high, making it impossible to make an exact statement about the actual concentration [14]. Another study shows that the concentration of alcohols present during the storage of meat increases in line with rising temperatures, irrespective of the storage atmosphere [82]. Refs. [14,94] show a dependency between alcohol formation and temperature, whereby an increase in temperature is accompanied by a proportional increase in alcohol formation. Nevertheless, additional research is required to substantiate this assertion.

#### 4.2.2. Atmosphere

Alcohols are produced by spoilage microorganisms in both aerobic and anaerobic conditions [1] Nevertheless, a study of the evolution of ethanol during storage at 4 °C in beef, poultry, and pork demonstrates an absence of a discernible trend in ethanol concentration across all three types of meat under aerobic conditions [98] and in beef also under vacuum [46,104]. Under vacuum conditions, the presence of heterofermentative lactic acid bacteria in beef and poultry may be indicated by an increase in ethanol concentration. (LAB) [98]. These observations correspond to the findings of a study of beef and pork, which indicates that ethanol formation is marginally higher under aerobic atmospheres than under CO_2_ or N_2_ packaging [97]. Additionally, elevated oxygen levels in MAP packaging is linked to an increased concentration of 1-octen-3-ol, 1-pentanol, hexanaldehyde, and 2-pentanone (pentane-2-one), which can be attributed to heightened lipid oxidation [87]. Consequently, a limiting factor for the utilization of alcohols as degradation products is their dependence on the O_2_ content of the atmosphere during storage. To illustrate, a study on beef storage at 4 °C under MAP (70% O_2_, 20% CO_2_, 10% N_2_) demonstrates the impact of O_2_ on the formation of various alcohols. The study identifies a notable increase in the concentration of alcohols, with 2-methylbutanol and 3-methylbutanol, 1-pentanol, and 1-hexanol emerging as potential degradation products in beef [86]. Given the contradictory statements, it is not possible to provide a definitive account of the formation and change in concentration of alcohols in relation to the packaging atmosphere.

#### 4.2.3. Meat Type

The formation of alcohols during spoilage is demonstrated in a variety of meat types in different studies. While 3-methylbutan-1-ol is identified as a potential degradation product in air-stored beef at 4 °C [104], ethanol shows a significant, though not robust, correlation with the microorganism concentration in the early stages of spoilage of chicken breast meat stored aerobically at varying temperatures (4, 10, 21 °C) [101]. A further study has revealed disparities in the evolution of alcohols during storage at 4 °C, both aerobically and under vacuum, of pork, beef, and poultry. While the alcohol concentration remains unaltered in pork and poultry, it increases in beef [98]. Given that alcohols are implicated in the spoilage of diverse types of meat, they possess the potential to be utilized as degradation products for various types of meat. Nevertheless, no single alcohol is identified in the literature as being particularly suitable.

### 4.3. Ketones

The formation of ketones is a consequence of microbial or chemical deterioration, occurring as a result of the breakdown of fats [1,90]. The presence of ketones is indicative of the activity of Gram-negative (e.g., *Pseudomonas*, *Shewanella* and *Moraxella*) or Gram-positive bacteria (*Carnobacterium* spp.). These bacteria are capable of producing ketones as a byproduct of alkane degradation or alcohol dehydrogenation [11,42,105,106]. Although ketones like 2-pentanone, 2,3-pentanedione (pentane-2,3-dione), 2,3-octanedione (octane-2,3-dione), and 3,5-octanedione (octane-3,5-dione) are associated with off-flavors [86]. Despite this, no specific ketone has been identified as a reliable indicator of meat spoilage [13]. The primary ketones responsible for the off-flavor of meat are acetoin (3-hydroxybutan-2-one) and diacetyl (butan-2,3-dione). With respect to bacterial production, *Brochothrix thermosphacta* has the capacity to produce acetoin [1]. Other ketones are also found in different types of meat, e.g., 2-butanone (butan-2-one), acetoin and diacetyl are commonly found in fresh pork [102] and acetone (propan-2-one) whose concentration remains constant during poultry storage at 1.5 ± 0.2 mg/m^3^. The acetoin concentration does not follow a clear trend, initially demonstrating a sharp increase from 5–8 mg/mg^3^ (between days 3 and 9 of storage), followed by a sharp decrease to 0–2 mg/m^3^. The formation of acetoin is associated with the metabolic activity of *Brochothrix thermosphacta*, *Carnobacterium* spp. and *Lactobacillus* spp. Additionally, a decrease in acetoin may occur concurrently with the potential formation of diacetyl [96]. A study of acetoin and diacetyl formation in pork at 4 °C reveals that acetoin increases rapidly, reaching 50 mg based on one litter of packaging volume on day 3. Thereafter, it decreases to 10 mg on day 4 and then increases again to almost 400 mg on day 6 [107]. This phenomenon is similar to that previously reported by [106]. In contrast to the aforementioned studies on chicken meat, the presence of acetoin is not indicated by [84]. In conclusion, the typical ketones, including acetone, acetoin, 2-butanone, and diacetyl, do not demonstrate a statistically significant positive correlation with meat spoilage.

#### 4.3.1. Temperature

The formation of ketones depends on the storage temperature. The concentration of diacetyl and acetoin is significantly higher at 10 °C than at 4 °C [14]. In the context of air, an increase in ketone concentration is indicated by a rise in temperature, which cannot be confirmed under MAP conditions [82]. Therefore, it appears that the formation of ketones is dependent on temperature. However, it can be reasonably deduced that the packaging atmosphere plays a more decisive role.

#### 4.3.2. Atmosphere

No general correlation has been identified between the influence of different atmospheres and the formation of ketones during spoilage. In contrast, there are ketones that are produced irrespective of the prevailing atmosphere, while others are produced by microorganisms only in specific atmospheric conditions [103]. Additionally, a study reports that packaging atmosphere shows an effect on ketone formation. In this study, acetoin and diacetyl are identified as possible degradation products in air-stored beef, while 2-butanone is additionally identified in vacuum-packed beef [108]. The majority of *Carnobacterium maltaromaticum* strains have been observed to produce acetoin in air and/or vacuum packaging. In contrast, almost none of the strains are able to form 2-heptanone (heptan-2-one) and 3-octanone (octan-3-one) under vacuum atmospheres. However, the majority of the strains are capable of doing so in air [103]. In addition to the microorganisms *Pseudomonas putida*, *Lactobacillus sakei*, *Pseudomonas fragi*, and *Leuconostoc mesenteroides* [95], and *Carnobacterium maltaromaticum* [109], the presence of ketones is also associated with *Lactobacillus*. This is shown by the storage of beef at 4 °C under vacuum [46]. Furthermore, a study examining the storage of beef at 4 °C under different atmospheres identifies correlations between the presence of acetoin and the growth of *Brochothrix thermosphacta* and *Pseudomonas* spp. under MAP and in bacteriocin-activated antimicrobial packaging [42]. Another study, also on beef storage at 4 °C under MAP (70% O_2_, 20% CO_2_, 10% N_2_), shows a significant increase in ketones during the storage and suggests the utilization of 2,3-octanedione and 3,5-octanedione, among other potential degradation products, for beef [86]. The existing literature does not present a unified perspective on the extent to which the atmosphere exerts an influence on ketones. Some studies indicate that certain ketones are formed with greater frequency in a vacuum, while others suggest that the presence of oxygen (O_2_) is a significant factor. Additionally, there are observations indicating that certain ketones are not influenced by the atmosphere.

#### 4.3.3. Meat Type

The formation of ketones is subject to different dynamics, depending on the type of meat in question [14]. With regard to pork, research indicates that storage at 4 °C under a high oxygen atmosphere results in an increase in the concentration of acetoin, diacetyl, and 3-methyl-1-butanol during the spoilage process [87]. In pork inoculated with *Pseudomonas putida*, *Lactobacillus sakei*, *Pseudomonas fragi*, and *Leuconostoc mesenteroides* and stored aerobically at 4 and 10 °C, the presence of ketones can also be indicated in the headspace of the packaging of pork that has undergone spoilage. Nevertheless, the presence of acetoin is also indicated in the headspace of packaging used for fresh pork [83]. The relationship between ketones and pork spoilage at 4 °C under MAP (70% O_2_, 30% CO_2_), is also corroborated by another study, wherein multiple ketones were identified over the course of a 12-day storage period. The data indicate an increasing rise for both acetone and acetoin, with acetone exhibiting a relatively high odor threshold and acetoin a low one. An increase in concentration up to day 6 and subsequent decrease up to day 12 can be determined for 2-butanone. In contrast, 2,3-butanedione (butane-2,3-dione) is not indicated until the end of the storage period [110]. A comparable phenomenon is observed with 2,3-Butanedione in the context of beef storage at 4 °C, under aerobic and vacuum conditions. Following an initial increase, a subsequent decrease in the ketone concentration is evident [108]. In a contrasting observation, the storage of poultry meat at 4 °C and MAP (40% CO_2_, 30% N_2_, 30% O_2_) reveals an increase in the concentration of 2-butanone until the conclusion of the 15-day storage period, with no subsequent decline in concentration [96]. A review of the scientific literature reveals a lack of consensus regarding the formation of ketones in diverse types of meat. A number of ketones have been found to exhibit elevated levels in pork samples (acetone, acetoin, diacetyl, and 3-methyl-1-butanol), whereas in poultry meat samples, 2,3-butanone is the predominant ketone present at elevated concentrations. No discernible trend is identified with respect to beef, and no pertinent literature is uncovered for other types of meat.

### 4.4. Sulfur Compounds

The presence of sulfides is among the earliest indications of meat spoilage [6]. This observation is a consequence of the primary source of sulfur compounds being the degradation sulfur-containing amino acids [111], including methionine ((2S)-2-amino-4-methylsulfanylbutanoic acid) and cysteine ((2R)-2-amino-3-sulfanylpropanoic acid) [10]. The concentration of sulfur compounds increases with storage time [98]. The most prevalent sulfur compound in spoiled meat is dimethyl sulfide, followed by dimethyl disulfide (methylsulphonylmethane), dimethyl trisulfide, and methyl thioacetate [11,112]. In contrast with the findings of [11,113], a study in which pork is inoculated with various spoilage microorganisms (*Pseudomonas fragi*, *Lactobacillus sakei*, *Pseudomonas putida* and *Leuconostoc mesenteroides*) and stored in air at 4 and 10 °C, the results show that dimethyl sulfide is mainly indicated in the fresh pork samples. In contrast, the presence of other substances, including alcohols, ketones, and esters, are identified in spoiled pork samples [83], which calls into question the use of dimethyl sulfide as a potential spoilage indicator. The hypothesis that dimethyl sulfide is a degradation product is also refuted by the finding that the concentration of dimethyl sulfide decreases with increasing storage time [83,109], which is in contrast to the findings of [98]. Another sulfide mentioned in the context of meat spoilage is hydrogen sulfide [105], which is formed by the enzymatic desulphidation of cysteine [114]. The presence of sulfur compounds such as dimethyl sulfide, dimethyl disulfide or dimethyl trisulfide, can cause unpleasant odors like petrol, rotten, fishy or sulfurous odors. Furthermore, sulfur compounds contribute to malodor and play a crucial role in the off-flavor of stored meat [115]. A comparison of Pearson correlation coefficients also shows that the linear correlations of sulfides with total viable count (TVC) are comparatively weaker (0.23–0.58) than those of alcohols (0.55–0.66) or free fatty acids (0.44–0.73), and also in comparison with TVC [100]. Nevertheless, sulfur compounds are indicated during meat spoilage [115,116], which suggests that they may still be regarded as potential degradation products.

#### 4.4.1. Temperature

Dimethyl sulfide concentration increases with prolonged storage time, temperature and microbial counts [117]. In various studies examining the storage of meat, sulfur compounds are identified at disparate temperatures, including 4 °C [116] and room temperature [111]. In the development of a dye to indicate the spoilage of poultry meat by color change due to the presence of volatile organic compounds in the headspace of the package. The study has examined the effect of room temperature and refrigerator temperature on consumer. In both cases, the color change occurs under the presence of sulfur compounds [115], which lends to support to the use of dimethyl sulfide as a degradation product for poultry meat. A correlation between the increase in dimethyl disulfide concentration and temperature can be observed [96]. The concentration of dimethyl disulfide increases with increasing temperature [117]. The available evidence indicates that sulfur compounds are present at varying temperatures, suggesting their potential as degradation products for monitoring meat spoilage. However, there is a paucity of literature examining the temperature dependence of sulfur compounds.

#### 4.4.2. Atmosphere

Sulfur compounds are mainly produced by *Enterobacteriaceae*, but also by *Pseudomonas* spp. *Enterobacteriaceae* grow in both aerobic and anaerobic conditions and are involved in meat spoilage [118]. For example, hydrogen sulfide can be used to indicate a variety of *Enterobacteriaceae* and is considered a possible indicator of meat spoilage under aerobic conditions [91]. Sulfur compounds are also indicated under aerobic conditions in association with *Pseudomonas* spp., while no indication occurs under MAP (70% O_2_, 30% CO_2_ and 30% CO_2_, 70% N_2_) [116]. Contrary to the statement of [119], the studies of [45,106] report that dimethyl sulfides can be indicated under MAP atmospheres (40% CO_2_, 30% N_2_, 30% O_2_ and 60% O_2_, 40% CO_2_) [42,96]. In contrast to the constant increase in the concentration of dimethyl sulfide under these conditions, carbon disulfide initially shows a decrease and a non-significant increase only on the last two days of measurement and is therefore not suitable as an indicator of meat spoilage [96]. This conclusion cannot be universally applied to the entire category of sulfur compounds. In the case of a developed indicator for monitoring the freshness of chicken meat, sulfur compounds have been observed to indicate at 6 °C in MAP, three-to-four days after the expiration of the best-before date. Consequently, this indicator is deemed suitable for indicating chicken meat spoilage under MAP packaging [120]. Furthermore, studies indicate the presence of sulfur compounds during meat storage under aerobic atmospheres, including 2-ethyl-1-hexanol [42], dimethyl trisulfide, and dimethyl sulfide [111]. In comparison to other degradation products such as acetoin with a concentration of 215 ppm in air, the sulfur-containing compounds are only indicated at extremely low levels, ranging from 0.5 to 2.96 ppm in air [42]. Despite the formation of sulfur compounds under disparate atmospheric conditions, it is challenging to identify a singular substance as the optimal degradation product due to discrepancies in the literature.

#### 4.4.3. Meat Type

Three studies report that dimethyl sulfide can be indicated in both poultry and beef [96,111,112]. In addition, other studies report the observation of sulfur compounds in pork [116] and in poultry [115]. It is therefore evident that sulfur compounds may be present in a range of meat types, also mentioned by [14], which elucidates that the formation of specific sulfur compounds is contingent upon the type of meat in question.

For instance, the concentration of dimethyl sulfide is known to increase when beef and poultry meat are stored under MAP (30% CO_2_, 70% O_2_) conditions and subsequently exhibits a decline in beef samples only. Nevertheless, the measured concentration of dimethyl sulfide in the packaging headspace is below 25 pp, which is a very low level [14].

It can therefore be surmised that sulfur compounds represent a less suitable option as degradation products within the headspace of intelligent packaging.

### 4.5. Esters

Esters are formed by the esterification of organic acids and alcohols and are associated with fruity, sweet or ethereal odors. The main ester producer is *Pseudomonas fragi* [11,14]. Nevertheless, the presence of *Pseudomonas fragi* is not generally associated with high ester formation during spoilage [106]. In this regard, the utilization of esters to indicate the presence of meat spoilage is not consistently reliable. Methyl ester is reported as the first degradation product [6]. Other esters, including ethyl acetate, ethyl butanoate, ethyl 3-methyl butanoate, ethyl octanoate, ethyl hexanoate, and ethyl decanoate, are formed later in the meat spoilage process [1,11].

#### 4.5.1. Temperature

Below a temperature of −1.5 °C, various bacteria such as LAB and *Enterobacteriaceae* show reduced growth, as they cannot use their metabolism properly at this temperature [15]. The same applies to psychrotolerant and mesophilic bacteria above 7 °C. Above this temperature, their metabolism does not function sufficiently to produce degradation products, such as esters [118]. However, several studies show that at least small amounts of esters can be indicated even above 7 °C [83,87,101]. Despite this, it is reasonable to assume that ester formation is strongest between −1.5 °C and 7 °C, which is exactly the recommended core temperature for meat of <5 °C in USA [60] or <7 °C in Europe [61,62].

#### 4.5.2. Atmosphere

The packaging atmosphere of stored meat exerts a significant influence on the formation of esters, with *Pseudomonas* spp. exhibiting a marked prevalence under aerobic conditions [41]. This is also confirmed by [11] who states that esters are mainly formed in fresh meat stored under aerobic conditions [11]. Ethyl esters can also occur under MAP (60% O_2_, 40% CO_2_) atmospheres, but are more commonly found under aerobic conditions. In the aforementioned atmospheres, it is not only *Pseudomonas fragi*, but other species of *Pseudomonas* spp. as well [85]. Two studies show that the indication of esters such as ethyl acetate during meat storage is possible under MAP (60–80% O_2_, 20–40% CO_2_) [87] as well as under air atmospheres. Given the low concentration (<peak area × 10^4^), the study further considers other degradation products, including alcohols, but not esters [101]. Nevertheless, none of the aforementioned studies propose esters as a degradation product for meat, in contrast to [121], which suggests esters as a potential degradation product for the indication of food spoilage. However, [122] does not differentiate between the various substances, such as biogenic amines (BA), alcohols or esters, that may be most suitable for meat [122]. The aforementioned studies indicate that esters are preferentially formed under aerobic conditions, although they can sometimes also be indicated under MAP atmospheres with O_2_. It may thus be postulated that the utilization of esters as degradation products is confined to packaging configurations with oxygen.

#### 4.5.3. Meat Type

Some esters, including ethyl acetate, ethyl propionate, ethyl butyrate and ethyl hexanoate can potentially be considered as characteristic compounds of fresh pork. A negative correlation, such as decrease over storage time, may be indicated for these esters [90]. Two other studies also report the presence of esters during pork storage. One study reports the presence of ethyl acetate [87], while the other describes esters in general and initially notes an increase in concentration and subsequently a decrease with storage time. [83]. In addition to pork, esters can also be indicated in chicken [101]. While the literature does make mention of the presence of esters in pork and chicken meat, no statements have been made regarding the presence of these compounds in other meat types, such as beef. Therefore, it is not possible to state that esters can be used universally to indicate the spoilage of different types of meat.

### 4.6. Carboxylic Acids

The hydrolysis of triglycerides and phospholipids results in the formation of volatile fatty acids [1], including carboxylic acids. Other possible pathways for the production of carboxylic acids are the degradation of amino acids or the oxidation of ketones, esters and aldehydes [123]. *Brochothrix thermosphacta* and *Carnobacterium* spp. are associated with the production of volatile fatty acids [123]. Other microorganisms that are known to produce acids are lactic acid bacteria (LAB), which are mainly responsible for the formation of lactic acid, but can also form acetic acid and other acids during storage [39,124]. Acetic acid is described as one of the most promising volatile degradation product for chicken meat. Nevertheless, propanoic acid, 2-methylbutanoic acid, 4-methylpentanoic acid and butanoic acid are also indicated in chicken meat, which is stored at 21 °C for three days [111]. Thiobarbituric acid (2-sulfanylidene-1,3-diazinane-4,6-dione) is also employed in the assessment of the sensory shelf life of foodstuffs, and is frequently utilized to quantify the extent of lipid oxidation [125].

#### 4.6.1. Temperature

The concentration of carboxylic acids formed is influenced by the duration and temperature of meat storage. This results in varying concentrations of carboxylic acids (specifically, octanoic acid and butanoic acid) during the storage of beef at temperatures of −2 °C and 4 °C. A comparison of different studies supports this assertion. Carboxylic acids is indicated during meat storage at a range of different temperatures including −3.5 °C [88], 4 °C [42,104,119], 6 °C [47] up to 21 °C [111]. At lower temperatures up to 6 °C, the formation of hexanoic acid [11], 3-methylpentanoic, pentanoic acid, octanoic, nonanoic, decanoic and oleic acids [119] is indicated. At higher temperatures (21 °C), propanoic acid, 2-methylbutanoic acid, 4-methylpentanoic acid is indicated [111]. The formation of acetic acid and butanoic acid occurs at temperatures that are both cooler and warmer [104,111], than those affecting the carboxylic acids mentioned previously, and thus are less affected by temperature. It can be reasonably concluded that acetic acid and butanoic acid are the most suitable degradation products for universal use.

#### 4.6.2. Atmosphere

The following fatty acids can be identified under different storage atmospheres: acetic acid [11], butanoic acid [42], and hexanoic acids [47] The formation of branched fatty acids, including 2- and 3-methylbutanoic acid, is exclusively observed under aerobic conditions [11]. In both vacuum-packed meat and under aerobic conditions, acetic, butanoic, and pentanoic acids are identified. As storage time increases, the concentration of carboxylic acids rises, resulting in perceptible changes in sensory properties. These changes are observed after seven days under aerobic conditions and after nine days under vacuum [104]. Such sensory alterations encompass, for instance, the occurrence of off-odors, which are attributable to the release of acetic acid and butanoic acid during the storage of beef [108]. In vacuum-packed meat, butanoic acid is produced by LAB via the degradation of amino acids by the Stickland reaction or by *Clostridia* via fermentative butanoic acid metabolism [42,91,113]. In aerobic conditions, *Pseudomonads* spp. are capable of forming gluconic acids specifically ((2R,3S,4R,5R)-2,3,4,5,6-pentahydroxyhexanoic acid) and 2-oxogluconic acids through the oxidation of glucose [126]. Additionally, carboxylic acids like hexanoic [47] and butanoic acid [11] are formed under MAP (80% O_2_, 20% CO_2_). Furthermore, pentanoic acid, n-heptanoic acid, n-octanoic acid, n-nonanoic acid, n-dodecanoic acid, propionic acid (propanoic acid) and isovaleric acid (3-methylbutanoic acid) are also indicated under MAP (high O_2_). In particular, n-octanoic acid, n-nonanoic acid, and pentanoic acid are prevalent, particularly in meat that has been packaged aerobically [88]. Furthermore, the study of the effects of different atmospheres on ground meat revealed an oxygen dependence of carboxylic acids. The study reveals that, in the majority of instances, the acetic acid concentration is higher in oxygen-rich atmospheres than in other atmospheres [97]. The existing literature indicates that certain carboxylic acids, such as acetic acid, are formed under different atmospheric conditions. This evidence supports the argument that these acids can be utilized as degradation products. However, the oxidation of acids that occurs when glucose is removed, as well as the varying glucose content of meat [91], are consequences that render these acids unsuitable as indicators of spoilage.

#### 4.6.3. Meat Type

Carboxylic acids are present in a range of meat types. Some studies demonstrate the presence of carboxylic acids in stored chicken [88,111,119], while others show that carboxylic acids also occur when storing beef [42,47,104]. A 45-day storage of beef at 4 °C under different atmospheres (air, MAP, vacuum, antimicrobial bacteriocin) shows a correlation between butanoic acid and the growth of lactic acid bacteria (LAB, with butanoic acid concentration increasing with increasing LAB concentration [42]. LAB are among the microorganisms that are most responsible for the spoilage of meat of various types [11,127]. In chicken, propanoic acid, 2-methylbutanoic acid, 4-methylpentanoic acid [111] 3-methylpentanoic, octanoic, nonanoic, decanoic and oleic acids [119] are indicated. Meanwhile, hexanoic acid is indicated in beef [47]. In both meat types, chicken and beef, acetic, butanoic [104,111], and pentanoic acids [104,119] are indicated. It is evidenced that carboxylic acids are formed during the spoilage of beef and chicken. Currently, however, there is a paucity of scientific literature pertaining to the formation of these acids in other types of meat.

### 4.7. Biogenic Amines

Polyamines, spermine (N,N′-bis(3-aminopropyl)butane-1,4-diamine) and spermidine (N′-(3-aminopropyl)butane-1,4-diamine) are naturally present in fresh meat. In contrast, biogenic amines (BAs) such as histamine (2-(1H-imidazol-5-yl)ethanamine), putrescine (butane-1,4-diamine), tyramine (4-(2-aminoethyl)phenol), tryptamine (2-(1H-indol-3-yl)ethanamine), and *β*-phenylethylamine (azane) are formed during storage [128]. BAs are the result of microbial activity where amino acid decarboxylases generate them from free amino acids (FAA) [129], therefore they could be used as bacteriological quality indicators [121]. BAs have already been proposed as an indicator of spoilage [43] or as a useful tool for monitoring meat quality and safety [130]. In particular, putrescine, histamine, cadaverine and tyramine have a high potential for indicating meat spoilage [29].

The formation of BAs is dependent on a number of factors that influence the FAA content and the activity amino acid decarboxylases in meat [130]. These factors include the composition of the meat and the characteristics of the microbial growth, in addition to the processing, preservation and storage conditions [130,131,132,133]. The spectrum of BAs produced is contingent upon the availability of precursors in the form of FAA, as well as the composition of the microbial flora. The discrepancies in FAA levels may be attributed, at least in part, to the differing proteolytic and amino-genic capabilities of the specific microbiota [134]. The formation of BAs increases at higher bacterial loads beginning at a concentration of log 6 CFU/cm^2^ [135]. Therefore, the formation of BAs is not linearly correlated with the number of microorganisms present, but there is a slight time lag between content of BAs and microbial growth [17]. A study of 50 poultry-associated bacterial strains (25 *Pseudomonas*, 13 *Salmonella*, and 12 *Listeria*) shows that different strains do not necessarily produce an identical profile of degradation products. Cadaverine (pentane-1,5-diamine), putrescine, phenylethylamine, histamine and tyramine are formed by all *Salmonella* strains tested. The formation of cadaverine, putrescine, and phenylethylamine is observed in over 90%. Additionally, the studied *Pseudomonas* strains demonstrate the production of histamine (60%), tryptamine (36%) and tyramine (16%). The *Listeria* strains do not form BAs [136]. Cadaverine is also primarily associated with *Enterobacteriaceae* and tyramine, putrescine, histamine and cadaverine to a lower extent with *Pseudomoas* spp., *Brochothrix thermosphacta* and *Pychrotrophs* [137]. Ref. [138] also found correlations between the amount of *Enterobacteriaceae* present with cadaverine, and between *Pseudomonas* spp. and *Brochothrix thermosphacta* with tyramine, and to a smaller extent with histamine and cadaverine [139]. In addition to a correlation between *Enterobacteriaceae* and aerobic mesophilic bacteria with cadaverine, a high positive correlation between putrescine and these microorganisms can also be observed. This implies that an increase in the concentration of BAs is associated with an increase in the number of microorganisms [121]. Furthermore, amino acids are metabolized by lactic acid bacteria (LAB), resulting in the production of biogenic amines, including tyramine, spermine, histamine, and putrescine [118]. It can thus be concluded that amines are dependent on the microorganisms involved in the process of meat spoilage. The presence of *Enterobacteriaceae* and *Pseudomonas* spp. in particular leads to an increase in the concentration of cadaverine, putrescine, tyramine and histamine.

BAs are also affected by different storage conditions [133]. For instance, the concentration of tyramine and cadaverine demonstrates a pronounced correlation with storage duration, exhibiting an increase in concentration with prolonged storage. In contrast, the effects on spermidine and putrescine are less pronounced [137]. Likewise, the pH value shows a pronounced effect on the formation of BAs [130].

#### 4.7.1. Temperature

The formation of BAs is influenced by both storage temperature and time. An increase in temperature, whether gradual or abrupt, has the effect of stimulating microbial growth. Similarly, the concentration of BAs is known to rise in response to elevated temperatures and extended storage time. Therefore, the concentrations of putrescine and cadaverine in poultry are also influenced by storage temperature [140]. Furthermore, histamine demonstrates a temperature-dependent behavior, exhibiting a concentration increase at temperatures above 4.9 °C, as opposed to a concentration decrease at temperatures below 18 °C [141]. Refs. [142,143] also conclude that the total amount of BAs is lower when meat is stored at lower temperatures (below 6 °C) [142,143]. Higher BAs concentrations are found at higher temperatures. In Addition, the influence of temperature on the formation of BAs in relation to the type of meat confirms that the temperature influence is most pronounced in poultry, in comparison to beef and pork. [144] Consequently, the concentration of BAs demonstrates a pronounced correlation with temperature, with an increase in concentration observed as temperature rises.

#### 4.7.2. Atmosphere

The atmosphere utilized for packaging can impact the qualitative and quantitative formation of Bas [145]. The study [46] on the effects of aerobic and anaerobic atmospheres on BAs at 4 °C confirms the observations of [146], who also show a significant influence of the atmosphere on the formation of BAs, especially putrescine and cadaverine [46,146]. In this regard, the concentration of these two BAs is higher under aerobic conditions than under MAP (30% CO_2_, 70% N_2_), which may be attributed to the enhanced growth characteristics of *Pseudomonas* spp. in this atmosphere [43,147]. The influence of *Pseudomonas* spp. on the formation of BAs is related to the decreasing availability of glucose. As the number of bacteria increases, the available glucose becomes insufficient, resulting in the subsequent degradation of amino acids and proteins into BAs and sulfides [39]. This correlates with the finding that the levels of putrescine and cadaverine increase linearly with storage time [43]. Facultative *Enterobacteriaceae* can also grow under all atmospheric atmospheres, with or without the presence of O_2_. *Enterobacteriaceae* growth leads to increased formation of cadaverine and putrescine, tyramine and histamine [148,149]. For example, the formation of putrescine and cadaverine in pork meat increases in all MAP atmospheres, whereas tyramine shows only minor increases [139]. However, less putrescine and cadaverine is formed in chicken tile under high O_2_ MAP (75% O_2_, 25% CO_2_) than under air or vacuum [150]. For vacuum-packed beef, the formation of BAs as a function of microbial load is confirmed [135]. The levels of other BAs, such as spermidine and spermine in pork, exhibit minimal variation in response to the different atmospheres [139]. Additionally, a reduction in these two BAs may occur in chicken meat [43]. The results demonstrate that, in particular, cadaverine and putrescine, but also tyramine and histamine, are promising degradation products under different conditions. In addition to the aforementioned increasing BAs, there are spermidine and spermine that do not demonstrate significant concentration changes during processing and storage [107,146,148].

#### 4.7.3. Meat Type

The formation of BAs is influenced by the conditions under which raw meat is handled. The different compositions of FAA that are dependent on the type of meat may result in either differentiating or unifying effects on red or white meat, respectively [129,131]. In a study examining the storage of meat, higher levels of cadaverine are observed in both red and white meat. This phenomenon may be attributed to the elevated levels of the precursor lysine present in these meats. In this experiment, all BAs exhibited an increase in concentration earlier in white meat than in red meat. This can be attributed to the shorter muscle fibers in white meat, which are more susceptible to proteolytic enzyme attack [151]. Another study yields comparable outcomes, with accelerated and elevated formation observed in chicken, turkey, and pork. The aforementioned observations pertain to the total free amino acid precursors of BAs [17]. For chicken meat stored at 4 °C ± 1 °C for 17 days, cadaverine, putrescin, and tyramine are identified as the major BAs for indirect bacterial indicators [121]. Other bases, including spermidine and spermine, as well as trace amounts of putrescine, have been identified in unripened chicken meat that has been subsequently stored at temperatures ranging from 1 to 1–5.7 °C for 8 h and at −18 °C ± 1 °C for 89 days. During the 8-h storage, an increase in tyramine and histamine a there is a notable increase in the levels of tyramine and histamine [141]. However, histamine is more commonly associated with the degradation of fish freshness [139] than with meat. With regard to the various muscle groups within a given species, there is no significant divergence in the profile of BA formation. Only minor differences in the levels of cadaverine and putrescine are observed, which can be attributed to different microbial counts resulting from different contamination during cutting [152]. Another experiment shows higher levels of amines in the breast compared to the leg of chicken meat stored for 15 days [147]. Besides the indication in chicken, BAs can also be indicated in beef. As storage time increases, BAs concentrations also increase, especially of tyramine and cadaverine. At the outset of the storage period, the highest concentrations of tryptamine and spermine is observed. However, with prolonged storage, no notable alterations are discerned in these BAs and histamines. For putrescine, there is a significant increase in concentration after eight days, but not as much as for cadaverine and tyramine [137]. The more specific protein composition and softer structure of chicken meat render it more susceptible to the formation of BAs than pork or beef [153]. Therefore, chicken meat exhibits a more rapid deterioration due to BAs than beef and pork, and to a similar extent as lamb [17]. It can be reasonably concluded that, although BAs, particularly cadaverine and tyramine, are indicated in the spoilage of various meat types, they appear to be superior degradation products for chicken in comparison to other meat types. It is challenging to utilize these compounds as broad-spectrum degradation products.

#### 4.7.4. Biogenic Amine Index

As different BAs occur in different meats, the use of a biogenic amine index (BAI) may be helpful in assessing meat freshness [154]. An advantage is that the BAI is not influenced by microbial activity and tends to remain stable during storage [130]. Over time, a variety of calculation models for BAI in meat is reported, some of which are shown in Table 2.

The profile of the formed BAs is not identical for all types of meats and the different packaging concepts [17,151]. Accordingly, disparate calculation bases are necessary for disparate scenarios, as well as disparate limit values for the classification of freshness based on the BAI value achieved. In various studies, the BAI limits range from 50 mg/kg [147,154] to 96–101 mg/kg [43] with the latter representing the threshold for spoilage in the studied meat types. Although it is possible to use BAIs as quality indices and indicators of undesirable microbial activity in meat [131], BAIs also have their limitations. A low concentration of BAIs does not necessarily indicate microbiological quality, as not all spoilage or starter microorganisms are capable of decarboxylating free amino acids [156]. Furthermore, common food infections such as *Listeria* do not lead to the formation of amines [136]. The BAI is therefore restricted to the indication of spoilage and is not a suitable indicator of the general risk of infection.

### 4.8. Total Volatile Basic Nitrogen

TVB-N can be used to indicate the freshness of meat [146,157]. Nevertheless, the widespread application of TVB-N as a spoilage indicating degradation product is constrained by the inconsistency and scarcity of findings pertaining to the correlation between TVB-N content and the freshness of diverse types of meat, e.g., beef, pork, chicken, and fish [146]. The degradation of nitrogenous protein and non-protein compounds results in the formation of a range of microbial degradation products, including nucleotide catabolites and amino acids, which contribute to an increase in TVB-N content. This is indicative of meat spoilage [157]. It can be reasonably inferred that microorganisms are also responsible for elevated TVB-N levels, as evidenced by the proliferation of *Pseudomonas* spp., including *Pseudomonas taetrolens* and *Pseudomonas fragi* during the storage of beef under air atmospheres and at 4 °C [108]. Furthermore, TVB-N is linked to the decarboxylase activity of microorganisms with regard to amino acid during the storage period [158]. Additionally, a correlation between TVB-N in chicken meat and microbiological and sensory values can be demonstrated, sensory values are observed to decrease with increasing TVB-N content [159]. Moreover, [160] demonstrates that the concentrations of putrescine, cadaverine and tyramine in beef, pork and chicken have a strong positive correlation with the TVB-N content during storage. The concentration of putrescine, cadaverine and tyramine increases with increasing TVB-N content [161]. Consequently, the increase in TVB-N is also delayed, with an increase occurring after a few days of storage [147]. This phenomenon is attributed to the initial consumption of glycogen by putrefactive bacteria, which subsequently initiates a shift towards protein degradation, leading to an increase in TVB-N. The existing literature shows consistency regarding the impact of TVB-N on the microbiological and sensory characteristic of meat. However, there is a lack of consensus regarding the optimal TVB-N threshold, which is supposed to indicate the freshness of meat, but no uniform limit has been established [146].

#### 4.8.1. Temperature

A slight correlation between the TVB-N value and the temperature is discerned. TVB-N increases at a gradual rate at low temperatures, which is likely due to a delay in bacterial growth at lower temperatures [162]. A similar phenomenon is observed in a study examining the storage of beef at temperatures of 4 °C and 28 °C. At 4 °C (start value: 0.001% N) the initial TVB-N limit of 0.017% N is exceeded after seven days, whereas at 28 °C (start value: 0.010% N) this limit is reached after 8 h. Consequently, the rate of TVB-N increase is slower at lower temperature. [163] A number of studies have examined the impact of temperature on the concentration of TVB-N, with experiments conducted at a range of temperatures, including −18 °C [107], 3 °C [164], 4 °C [107,108], 5 °C [162] and 28 °C. The findings indicate that the content increases at a faster rate with increasing temperature, for example, the TVB-N limit of 15 mg/100 g, other factors being equal, is exceeded at −18 °C between 466 and 494 days, while at 4 °C it is already exceeded after five days [107]. This evidence supports the conclusion that the TVB-N content is temperature dependent and increases with rising temperatures.

#### 4.8.2. Atmosphere

The development of TVB-N compounds on chicken breast is highly dependent on the storage atmosphere. Observation of the evolution of TVB-N on chicken breast during different storage phases and in different atmospheres at 5 °C shows that in all atmospheres (A—0.03% CO_2_, 21% O_2_, 78% N_2_; B—50% CO_2_, 50% O_2_; C—100% CO_2_) there is an increase in TVB-N concentration during storage. On day 0, TVB-N levels are below 15 mg per 100 g and increase to 28.689 ± 0.15 (C), 30.831 ± 0.70 (B) and 40.475 ± 0.80 (A) mg per 100 g by day 9, depending on the atmosphere. With increasing CO_2_ content (50 and 100%), the magnitude of the TVB-N increase decreases [158]. Similar results can be indicated in turkey meat, where this effect is associated with inhibition of *Pseudomonas* spp. and *Enterobacteriaceae* under MAP atmospheres [2]. In general, studies are available on different atmospheres, air [108], vacuum [108,164] and MAP [158,164]. In a comparison between beef storage in air and vacuum-packed, it is indicated that the established TVB-N limit of 20 mg/100 g is reached faster in air (after nine days) than in vacuum (after 21 days) [108]. In contrast, the atmosphere does not appear to have a significant effect on TVB-N levels during turkey meat storage [164]. The TVB-N content thus increases with increasing O_2_ content and decreasing CO_2_ content and is thus dependent on the atmosphere. However, the dependence is not too strong, since in all the studies mentioned a comparable increase in TVB-N content occurred in all atmospheres.

#### 4.8.3. Meat Type

There is a diversity of recommendations pertaining to TVB-N spoilage limits, which are contingent upon the specific categories of fish, seafood, and meat in question. Consequently, it is not feasible to consider these recommendations as universally applicable [146]. The disparate recommendations are attributable to the variations in physiology, meat characteristics, and nutrient content among the diverse types of meat [157]. In a study on chicken and beef, a limit of 20 ± 2 mg/100 g is set, which is reached by chicken when beef is still at a value of 5 mg/100 g [165]. A further TVB-N limit is utilized to ascertain the spoilage point of pork, with an assumed value of 15 mg/100 g of meat, derived according to Chinese standard GB 2707-2016. The study reveals that the specified limit is reached after eight days storage of at 4 °C [166]. A further analysis of the spoilage of pork at the TVB-N limit of 15 mg/100 g indicates that this limit is exceeded after five days at 4 °C. The initial TVB-N level is 3 mg/100 g, and a rapid increase is indicated from day 3 onwards [107]. In particular, the more pronounced increase observed after a few days lends support to the suggestion that TVB-N may be employed as a degradation product. Other TVB-N limits are reported in the scientific literature, including 17 mg/100 g for beef [167], and 20–30 mg/100 g for beef and pork [158]. Moreover, there are studies on duck meat [157], seafood [159] and, turkey meat, although the TVB-N value for turkey meat appears to be questionable [143]. A further possibility, as demonstrated by the analysis of TVB-N content in chicken breast and thigh meat stored at 4 °C, is that different cuts of meat from the same species may exhibit differing TVB-N values. The samples exhibited disparate TVB-N levels at both day zero and day 15, with higher levels observed in the thigh than in the breast meat [147]. The TVB-N content can be utilized as a degradation product for diverse types of meat. While the permissible limits per type of meat and, in select instances, per individual piece of meat, do vary, they typically range from 15 mg/100 g [107,166] to 30 mg/100 g [158].

## 5. Discussion

The potential for intelligent packaging to reduce meat spoilage represents a promising avenue for future research. However, they are still in the developmental phase, which is why it is crucial to identify degradation products of meat spoilage in order to develop indicators for indicating meat spoilage in intelligent packaging. This review identifies potential degradation products and discusses them in detail. Table 3 provides an overview of the results of the review. Nevertheless, when looking at the individual conditions in relation to the respective degradation products, no general correlation can be established, as there are more promising (e.g., acetic acid in relation to the wide temperature range which it can be identified) and less promising (e.g., ethyl acetate, which is only mentioned in the literature in connection with pork, so it remains unclear whether it can also be identified in other types of meat) results for specific substances within a degradation product.

The implementation of a method for identifying instances of meat spoilage through the utilization of freshness indicators across a spectrum of food packaging requires the formation of degradation products across a range of temperatures, atmospheres, meat types and microbial species associated with meat spoilage. As detailed in chapter 3, these factors exert a considerable influence on the concentration of these degradation products.

The degradation products considered are formed via disparate mechanisms, yet they can all be generated during the process of meat spoilage, as evidenced by the various studies presented in chapter 4. These degradation products are mainly due to the presence of *Pseudomonas* (aldehydes, alcohols, ketones, sulfur compounds, esters, BAs, TVB-N), *Carnobacterium* spp. (aldehydes, alcohols, ketones, carboxylic acids), and/or *Enterobacteriaceae* (aldehydes, sulfur compounds, BAs, TVB-N), which are involved in the spoilage of meat. In order to assess the feasibility of indicating meat spoilage using freshness indicators, a range of factors influencing the formation and concentration of degradation products are taken into consideration. These influences include temperature, atmosphere, meat type, and the presence of meat spoilage microorganisms. A discussion of the results pertaining to the individual categories of degradation products is presented in the following sections.

### 5.1. Aldehydes

Not all aldehydes are formed by microorganisms (mainly *Pseudomonas* spp., *Carnobacterium* spp. and *Enterobacteriaceae* [11,88], e.g., hexanaldehyde can also be formed by non-microbial factors such as the hydrolysis of triglycerides [106,178], although it is among the most commonly found aldehydes in spoiled meat [11]. The aldehydes also formed by non-microbial factors are therefore unsuitable for indicating meat spoilage. However, some aldehydes such as 3-methylbutanal are directly linked to spoilage different meat types in various studies. Certainly, the atmosphere plays a limiting role in this respect. For example, 2-butenal and malonaldehydes are associated with aerobically packaged [95,169], acetaldehyde with vacuum packaged [95], and octanal, nonanal, 2 and 3-methylbutanal with MAP packaged [86] spoiled meat. Not only the atmosphere, but also the type of meat leads to the formation of different aldehydes, due to the different composition of fatty acids available for oxidation depending on the type of meat [88]. Temperature also has an effect on aldehyde concentration, which increases as the temperature rises [15]. Overall, the presence and type of aldehydes vary too much with meat type, atmosphere and (non-)presence of microorganisms in meat spoilage to be applicable for a general indicator of spoilage in different packaging concepts.

### 5.2. Alcohols

There are rather fewer recent studies [14,101] investigating alcohol as a degradation product in meat. Instead, the majority of studies (e.g., [174,179]) investigate alcohol more as an indicator producer of food spoilage. Since alcohols are formed by the breakdown of carbohydrates, oxidation and hydrolysis of fats, or reduction of ketones and aldehydes, their concentration increases only with prolonged storage [1,90] of seven [14] to 12 days [96]. Similarly, temperature also has an effect on alcohol formation, but studies disagree on whether formation increases with rising temperature [82] or whether alcohols can no longer be indicated at a higher temperature [14]. Microorganisms for alcohol production are mainly *Pseudomonas* spp. and *Carnobacterium* spp. [1], although alcohols are described as unsuitable as degradation products, as some of them (e.g., 1-octen-3-ol and 1-pentanol) occur in the presence and absence of microorganisms [14,87]. As with aldehydes, there is alcohol formed under different atmospheres for these degradation products. In general, alcohols appear to be formed mainly in the presence of O_2_ [87,97], although another study has found an increase or decrease in ethanol under aerobic and vacuum conditions, depending on the type of meat [95]. For chicken meat, ethanol seems to be a possible degradation product [101,180], but this correlation between ethanol and *Pseudomonas* spp. formation does not always seem to exist, which argues against the use of ethanol as a degradation product [14]. Thus, the studies on the development of alcohols during meat storage do not show a clear general trend, but depend on environmental influences or are contradictory depending on the study.

### 5.3. Ketones

Acetoin and diacetyl are considered the major ketones for off-flavors in packed meat [1], but are also present in fresh meat [102]. In addition, acetoin shows inconsistent increases and decreases in concentration during storage of poultry [96] and pork [107], with increases associated with *Brochothrix thermosphacta*, *Carnobacterium* spp. and *Lactobacillus* spp. and decreases associated with possible concomitant diacetyl formation [96]. Furthermore, ketone formation is associated with *Photobacterium* spp. [87]. Diacetyl shows an increase in concentration and subsequent decrease during chicken storage [84]. In addition to pork and poultry meat, ketones are also indicated in the spoilage of beef [46]. Thus, these typical ketones do not show a significant increasing correlation with meat spoilage. Furthermore, ketone formation increases with increasing temperature [14], but there are conflicting statements as to whether this effect is shown only under air [82] or also under MAP atmospheres [14]. In general, no clear correlations can be made with the behavior of ketone concentration and different storage atmospheres. For example, acetoin can be formed under air and under vacuum conditions [103], while another study indicates acetoin and diacetyl under air but not under vacuum conditions [108]. Overall, there are conflicting conclusions as to whether or not ketones can be used as an indicator of meat spoilage, like [14] denies ketones as an indicator of meat spoilage [14] while [98] indicates 2,3-octanedione and 3,5-octanedione, among others, as possible degradation products at least for beef [14,86].

### 5.4. Sulfur Compounds

Dimethyl sulfide is the most abundant sulfur compound in spoiled meat [11,112] and has already been identified as a possible degradation product in several studies [14,42,96,111,112]. Dimethyl sulfide increases with increasing temperature and storage time [117] and can be associated with spoilage of various meats, such as beef and poultry [96,111,112], and sulfur compounds are also indicated in spoiled pork [116]. Sulfur compounds are mainly produces in the presence of *Enterobacteriaceae* and *Pseudomonas*, which grow under aerobic and anaerobic conditions [118]. Thus, sulfur compounds are indicated under air conditions [42,111]. Inconsistencies exist in indicability under MAP, with some studies [42,96] indicating sulfur compounds under MAP and other studies not [42,116]. There have been initial developments of indicators to show the spoilage of chicken meat by sulfur compounds [115,120] and also that sulfur compounds are considered to be one of the first indicatable degradation products [6]. Sulfur compounds are not necessarily suitable as indicators of meat spoilage because they occur in somewhat lower concentrations than other degradation products such as alcohols, ketones [101] or esters [83,111]. Moreover, sulfur compounds can also be indicated in fresh meat [83] and show a decrease with progressive meat spoilage [109]. All these inconsistencies suggest that the use of sulfur compounds as an indicator of meat spoilage is rather inappropriate.

### 5.5. Esters

Although esters are also considered to be early indicators of meat spoilage [6], only one [122] of the studies identifies them as degradation products. The other studies show ester indication in pork under MAP [87] and chicken under air conditions, but only at low concentrations of <<peak area × 10^4^ [101]. Ambiguous results, such as a concentration increase and subsequent decrease [83] or negative correlations [90], are also present in the literature. Since esters are mostly formed by *Pseudomonas fragi*, ester formation usually occurs under aerobic conditions [11,85], which argues against their wide application in different packaging concepts. However, as esters are formed in the temperature range (−1.5 to 7 °C [127]) in which fresh meat should be stored, they are suitable as decomposition products to indicate meat spoilage at least in relation to the temperature.

### 5.6. Carboxylic Acids

Different carboxylic acids are formed depending on the storage temperature [181]. Hexanoic acid [11], 3-methylpentanoic acid, pentanoic acid, octanoic acid, nonanoic acid, decanoic acid and oleic acid [119] are formed at cooler temperatures (up to 6 °C) and propanoic acid, 2-methylbutanoic acid, 4-methylpentanoic acid are formed at warmer temperatures (21 °C) [111], although in reality meat is not stored at such high temperatures. Both apply to acetic acid and butanoic acid, which are formed at cooler and warmer temperatures [104,111]. Fatty acids that can be indicated under different atmospheres include acetic acid [11], butanoic acid [42], and hexanoic acids [47], while branched-chain fatty acids are formed only in the presence of air [11]. Different carboxylic acids can be determined in different meat types (chicken and beef), with acetic acid, butanoic acid [104,111] and pentanoic acid [104,119] indicated in both meat types. Of the carboxylic acids, acetic acid, butanoic acid, and pentanoic acid appear to be the main degradation products, as they are formed in at least two types of meat and in different atmospheres. Advantages of carboxylic acids as suitable degradation products to indicate meat spoilage are that they can be indicated in the headspace of a package [88] and in one study dominate other volatiles [119]. On the downside, acids self-oxidize when glucose is removed, and as meat has a variable glucose content [91], this argues against the use of acids as degradation products.

### 5.7. Biogenic Amines

Biogenic amines (BAs), especially putrescine, histamine, cadaverine and tyramine [29] have already been identified as possible degradation products [130], as their concentration increases with storage time [139,150], whereas for other BAs, such as spermine or spermidine, it remains constant [139] or even decreases [43]. However, contrary studies find no significant histamine increase [137,139]. The formation of BAs depends on several factors. These include the composition of the meat, its processing and preservation, the characteristics of the growing microorganisms and the storage conditions [130,131,132,133]. The BAs concentration increases with increasing temperature, especially above 6 °C [140,182] and shows a greater influence on poultry meat than on other types of meat, such as pork or beef [144]. Also depending on different bacterial strains, different BAs are formed [134]. Cadaverine is mainly associated with *Enterobacteriaceae* and tyramine, putrescine, histamine with *Pseudomoas* spp., *Brochothrix thermosphacta*, *pychrotrophs* [137] and lactic acid bacteria (LAB) [118]. Due to more favorable aerobically growth characteristics of *Pseudomonas* spp., higher concentrations of putrescine and cadaverine are indicated under aerobic conditions than under MAP [43,147]. However, an increase in the concentration of BAs is also evident under MAP [139]. Another study shows that an increase in concentration can be indicated not only in air, but also in a vacuum [150]. That BAs can be indicated in the spoilage of various meat types, such as chicken, turkey, pork, lamb, and beef [17], argues for their application as degradation products. Moreover, for broader application, the presence of different BAs can be combined to form a BAI. However, there is currently no single BAI, but a variety of different ones, as shown in Table 2. Despite some of the advantages mentioned, BAs also have the disadvantage that not all microorganisms can decarboxylate free amino acids, so a low BA concentration does not automatically indicate high microbiological quality [156]. The suitability of BAs as indicators of meat spoilage is therefore limited. If used, it should be noted that cadaverine, putrescine, and tyramine have the greatest potential and can therefore be used individually or combined in a BAI.

### 5.8. Total Volatile Basic Nitrogen

TVB-N content is mainly associated with *Pseudomonas* spp. and *Enterobacteriaceae* [90]. The researched literature [90,108,158] shows consistency regarding the effect of TVB-N on microbiological and sensory characteristics of meat. Furthermore, a slower TVB-N increase is evident at lower temperatures [162], and TVB-N concentration raises with increasing temperature [107,163]. TVB-N compounds are formed under different atmospheres, with less formation in aerobic packages [108,158]. Due to the different physiology, meat characteristics, and nutrients in different meat types, different TVB-N perishability limits, per meat type [146] or even piece of meat within a species [147], must be established. This makes a general application challenging, but an application is still conceivable if an individual TVB-N perishability limit is determined and set for each type of meat and perhaps also for each piece of meat within a type of meat. In the researched literature, the following limits are given for different meat types 20 mg/100 g for chicken [175], 17 mg/100 g [167], 20 mg/100 g [108] or 20–30 mg/100 g [158] for beef and 15 mg/100 g [107,166] or 20–30 mg/100 g [158] for pork. TVB-N compounds can thus find application as an indication of meat spoilage in different packaging concepts and for different types of meat. A possible application to indicate meat freshness has also been reported in the researched literature [146,157], with conflicting and limited results reported on the relationship between TVB-N content and meat freshness [146].

## 6. Conclusions

The objective of this review is to establish a basis for the search for optimal degradation products whose concentration correlates with the shelf life of meat, and to evaluate their potential for indicating meat spoilage. In order to achieve this objective, this review considers eight different degradation products.

In light of the results presented in this review, it can be concluded that an indication pathway via a single degradation product is a challenging proposition. While some degradation products (aldehydes, alcohols, ketones, sulfur compounds, esters) seem to be less suitable as spoilage indicators, others (carboxylic acids, BAs, TVB-N) demonstrate greater potential. Although the more suitable degradation products also have disadvantages, as previously discussed, individual substances in these groups can be identified as potential degradation products. These include acetic acid, butanoic acid, and pentanoic acid, which belong to the carboxylic acids, and cadaverine, putrescine, and tyramine, which are classified as BAs. Additionally, these three BAs can be utilized in combination as BAI.

The use of TVB-N has the greatest potential, but the spoilage limits of TVB-N need to be determined and standardized for widespread use. Additional research is also required to ascertain the potential of combinations of different degradation products. Prioritization should be given to combinations of carboxylic acids, BAs, and TVB-N, as these have the greatest potential for indicating meat spoilage using freshness indicators in intelligent packaging concepts.

Further research may be warranted regarding the following steps. A systematic analysis should be conducted to determine the role of intelligent packaging in improving the shelf life and safety of meat. This analysis should identify specific degradation products that correlate with meat spoilage, taking into account the various influencing factors. It would be beneficial to assess the potential integration of these degradation products into intelligent packaging concepts, with a view to ensuring their suitability for real-world applications. This could be achieved by investigating the influence of different factors, such as temperature, atmosphere, and meat type. Furthermore, it would be advantageous to gain a clear understanding of the mechanisms underlying meat spoilage and the effectiveness of different degradation products as indicators for intelligent packaging applications. Ultimately, this research could contribute to reducing food wastage and improving food safety by providing insights into the development of more accurate and consumer-friendly spoilage detection methods.

## Figures and Tables

**Table 1:** Overview of microorganisms mainly responsible for meat spoilage under aerobic, anaerobic and facultative anaerobic conditions.

Preferred Growth Atmospheres		
Aerobic Microorganisms	Anerobic Microorganisms	Facultative Anaerobic Microorganisms
*Pseudomonas* spp. [42,43,44,45,46,47,48] *Acinetobacter* spp. [42] *Psychrobacter* spp. [42] *Moraxella* spp. [42] Molds [36]	Lactic acid bacteria (e.g., *Lactobacillus*) [1,49,50,51] *Clostridium* spp. [53,54] ^b^ *Brochothrix* spp. [42,52] ^a/b^	*Enterobacteriaceae* [42,55] ^a^ Yeasts [55]

^a^: preferred occurrence in MAP [42,52,55]. ^b^: preferred occurrence in vacuum packaging [42,52,54,55].

**Table 2:** Examples of calculation models for the biogenic amines index (BAI) used in different studies to combine different BAs for assessing meat freshness more easily.

Calculation Models for the BAI	BAI Value above Which Meat Is Considered Spoiled	Meat Type	Atmosphere	References
Putrescine + cadaverine + tyramine + histamine	>50 mg/kg	Pork	No specification	[138,154]
		Chicken	Air, MAP (75% O_2_, 25% CO_2_), vacuum	[150]
		Different meat types	No specification	[142]
(histamine + putrescin + cadaverine): (1 + spermine + spermidine)	>10 mg/kg (considered as low quality)	Meat in general	No specification	[155]
Putrescine + cadaverine + tyramine	No specification	Turkey	MAP (different compositions)	[143]
		Chicken	Air, MAP (70% N_2_, 30% CO_2_)	[43]

**Table 3:** The following is a summary of the most significant findings of the review, with the objective of presenting a preliminary classification of the degradation products under consideration. Nevertheless, the possibility of the existence of additional degradation product categories or specific substances within these categories under the specified conditions cannot be ruled out. Moreover, no definitive correlations can be identified, as the formation of degradation products is contingent upon specific conditions and the presence of various substances.

Degradation Product	Related Microorganisms	Conditions in Which the Degradation Products Are (Frequently) Measured		
		Temperature	Atmosphere	Meat Type
Aldehydes	*Pseudomonas* spp. *Carnobacterium* spp. *Enterobacteriaceae* [11,90]	Increases with increasing temperature [15,94,101]	MAP (30–70% O_2_, 20–40% CO_2_, 10–30% N_2_) [98,106] Vacuum [168] Aerobic [103,168]	Beef [102] Pork [102] Poultry [102,107,169]
Alcohols	*Pseudomonas* spp. *Carnobacterium* spp. [1]	Various statements: increases with rising temperature [94] or indication at 4 °C, but not at higher temperatures (10 °C) [14]	Mainly aerobic [99,114] MAP with high amount of O_2_ (70% O_2_, 20% CO_2_, 10% N_2_) [98] Vacuum [168]	Beef [102] Chicken [111,170]
Ketones	*Brochothrix thermosphacta* *Carnobacterium* spp. *Lactobacillus* spp. [106] *Photobacterium* spp. [99]	Increases with increasing temperature [14]	MAP (70% O_2_, 20% CO_2_, 10% N_2_) [98] Vacuum [109] Aerobic [94]	Beef [49] Pork [91] Poultry [36,106],
Sulfur Compounds	*Enterobacteriaceae* Pseudomonas [127]	Increases with increasing temperature [126] Indication at 4 °C [119] Indication at room temperature [123]	MAP (30–60% O_2_, 40% CO_2_, 30% N_2_) [45,106] Aerobic [45,123]	Beef [106,113,123] Pork [119] Poultry [106,113,123]
Esters	*Pseudomonas fragi* [11,96] *Pseudomonas* spp. [96]	Mostly formed between—1.5 °C and 7 °C	MAP (60–80% O_2_, 20–40% CO_2_) [96,99] Aerobic [11,43,111]	Pork [95,99] Chicken [111]
Carboxylic acids	*Brochothrix thermosphacta* *Carnobacterium* spp. [131]	Indicated between—3.5 °C [98] and 21 °C [123]	MAP (80% O_2_, 20% CO_2_) [90] Aerobic [115,136] Vacuum [115] Increases with CO_2_ increase [136]	Beef [45,50,115,123,135] Chicken [90,115,123,135]
Biogenic amines	*Enterobacteriaceae* [138,145,154] *Pseudomonas* spp. [138,153] Lactic acid bacteria [127]	Increases with increasing temperature [142] Indicated below 6 °C [142,143] to—18 °C [155]	MAP (0–75% O_2_, 25–30% CO_2_, 0–70% N_2_) [46,145,146] Aerobic [46,146,161] Vacuum [161]	Beef [154,163] Pork [17] Poultry [17,145,146,155,163,164] Fish [138] Lamb [17]
Total volatile basic nitrogen	*Pseudomonas* spp. [120]	Indicated between—18 °C [91] and 28 °C [171] Increases faster with increasing temperatures [91]	MAP (0–50% O_2_, 0.03–100% CO_2_, 0–78% N_2_) [2,136,172] Aerobic [120] Vacuum [120,172]	Beef [136,173] Pork [91,136] Poultry [146,174,175] Seafood [176,177]

## Data Availability

The original contributions presented in the study are included in the article, further inquiries can be directed to the corresponding author.

## References

[1] Pellissery A.J., Vinayamohan P.G., Amalaradjou M.A.R., Venkitanarayanan K., Mandal P., Biswas A. (2020). Spoilage bacteria and meat quality. Meat Quality Analysis: Advanced Evaluation Methods, Techniques, and Technologies.

[2] Fraqueza M.J., Ferreira M.C., Barreto A.S. (2008). Spoilage of light (PSE-like) and dark turkey meat under aerobic or modified atmosphere package: Microbial indicators and their relationship with total volatile basic nitrogen. Br. Poult. Sci..

[3] Kaur M., Williams M., Bissett A., Ross T., Bowman J.P. (2021). Effect of abattoir, livestock species and storage temperature on bacterial community dynamics and sensory properties of vacuum packaged red meat. Food Microbiol..

[4] Rimbach G., Nagursky J., Erbersdobler H.F. (2015). Erbersdobler, Lebensmittel-Warenkunde für Einsteiger.

[5] Fresán U., Mejia M.A., Craig W.J., Jaceldo-Siegl K., Sabaté J. (2019). Meat Analogs from Different Protein Sources: A Comparison of Their Sustainability and Nutritional Content. Sustainability.

[6] Mandal P., Biswas A. (2020). Meat Quality Analysis: Advanced Evaluation Methods, Techniques, and Technologies.

[7] Sutherland J., Bøgh-Sørensen L., Zeuthen P. (2003). Modelling food spoilage. Woodhead Publishing in Food Science and Technology, Food Preservation Techniques.

[8] GEMET; Degradation Product. https://www.eionet.europa.eu/gemet/en/concept/2042.

[9] Zhou G.H., Xu X.L., Liu Y. (2010). Preservation technologies for fresh meat—A review. Meat Sci..

[10] Dave D., Ghaley A.E. (2011). Meat Spoilage Mechanisms and Preservation Techniques: A Critical Review. Am. J. Agric. Biol. Sci..

[11] Casaburi A., Piombino P., Nychas G.-J., Villani F., Ercolini D. (2015). Bacterial populations and the volatilome associated to meat spoilage. Food Microbiol..

[12] Regez P., Gallo L., Schmitt R.E., Schmidt-Lorenz W. (1988). Microbial Spoilage of Refrigerated Fresh Broilers. III. Effect of Storage Temperature on the Microbial Association of Poultry Carcasses. *Lebensmittel − Wissenschaft + Technologie = Food Science + Technology*. https://www.cabidigitallibrary.org/doi/full/10.5555/19891350480.

[13] (2005). 2073/2005 of 15 November 2005 on Microbiological Criteria for Foodstuffs.

[14] Franke C. (2018). Untersuchung der Dynamik Flüchtiger Organischer Verbindungen von Schutzgas-Verpacktem Fleisch als Grundlage für Intelligente Verpackungen.

[15] Borch E., Kant-Muermans M.L., Blixt Y. (1996). Bacterial spoilage of meat and cured meat products. Int. J. Food Microbiol..

[16] (2015). Sensory Analysis—Assessment (Determination and Verification) of the Shelf Life of Foodstuffs.

[17] Triki M., Herrero A.M., Jiménez-Colmenero F., Ruiz-Capillas C. (2018). Quality Assessment of Fresh Meat from Several Species Based on Free Amino Acid and Biogenic Amine Contents during Chilled Storage. Foods.

[18] Statista, Wie Häufig Treffen die Folgenden Gründe auf Sie zu, Wenn Sie Lebensmittel Wegwerfen?: (Mindestens Gelegentlich). https://de.statista.com/statistik/daten/studie/486235/umfrage/umfrage-zu-gruenden-fuer-das-wegwerfen-von-lebensmitteln-in-deutschland/.

[19] Schumann B., Schmid M. (2018). Packaging concepts for fresh and processed meat—Recent progresses. Innov. Food Sci. Emerg. Technol..

[20] Boukid F. (2022). Smart Food Packaging: An Umbrella Review of Scientific Publications. Coatings.

[21] EFSA (2009). Guidelines on submission of a dossier for safety evaluation by the EFSA of active or intelligent substances present in active and intelligent materials and articles intended to come into contact with food. EFS2.

[22] Chen S., Brahma S., Mackay J., Cao C., Aliakbarian B. (2020). The role of smart packaging system in food supply chain. J. Food Sci..

[23] (2009). COMMISSION REGULATION (EC) No 450/2009 of 29 May 2009 on Active and Intelligent Materials and Articles Intended to Come into Contact with Food: 450/2009. https://eur-lex.europa.eu/legal-content/EN/TXT/PDF/?uri=CELEX:32009R0450.

[24] Flores Y., Pelegrín C.J., Ramos M., Jiménez A., Garrigós M.C. (2021). Use of herbs and their bioactive compounds in active food packaging. Aromatic Herbs in Food.

[25] Kerry J.P., O’Grady M.N., Hogan S.A. (2006). Past, current and potential utilisation of active and intelligent packaging systems for meat and muscle-based products: A review. Meat Sci..

[26] Yam K.L., Takhistov P.T., Miltz J. (2005). Intelligent Packaging: Concepts and Applications. J. Food Sci..

[27] Müller P., Schmid M. (2019). Intelligent Packaging in the Food Sector: A Brief Overview. Foods.

[28] Kalpana S., Priyadarshini S.R., Leena M.M., Moses J.A., Anandharamakrishnan C. (2019). Intelligent packaging: Trends and applications in food systems. Trends Food Sci. Technol..

[29] Fletcher B., Mullane K., Platts P., Todd E., Power A., Roberts J., Chapman J., Cozzolino D., Chandra S. (2018). Advances in meat spoilage detection: A short focus on rapid methods and technologies. CyTA—J. Food.

[30] Cloak O.M., Duffy G., Sheridan J.J., Blair I.S., McDowell D.A. (2001). A survey on the incidence of Campylobacter spp. and the development of a surface adhesion polymerase chain reaction (SA-PCR) assay for the detection of Campylobacter jejuni in retail meat products. Food Microbiol..

[31] Ajaykumar V.J., Mandal P.K. (2020). Modern concept and detection of spoilage in meat and meat products. Meat Qual. Anal..

[32] Weng X., Luan X., Kong C., Chang Z., Li Y., Zhang S., Al-Majeed S., Xiao Y. (2020). A Comprehensive Method for Assessing Meat Freshness Using Fusing Electronic Nose, Computer Vision, and Artificial Tactile Technologies. J. Sens..

[33] Miller K., Reichert C.L., Schmid M. (2021). Biogenic Amine Detection Systems for Intelligent Packaging Concepts: Meat and Meat Products. Food Rev. Int..

[34] Horak I., Engelbrecht G., van Rensburg P.J.J., Claassens S. (2019). Microbial metabolomics: Essential definitions and the importance of cultivation conditions for utilizing Bacillus species as bionematicides. J. Appl. Microbiol..

[35] Lim L.-T. (2011). Active and Intelligent Packaging Materials. Comprehensive Biotechnology.

[36] Wang G.-Y., Wang H.-H., Han Y.-W., Xing T., Ye K.-P., Xu X.-L., Zhou G.-H. (2017). Evaluation of the spoilage potential of bacteria isolated from chilled chicken in vitro and in situ. Food Microbiol..

[37] Nychas G.-J.E., Skandamis P.N. (2005). Fresh Meat Spoilage and Modified Atmosphere Packaging (MAP).

[38] Guerrero I., Chabela L.P., Robinson R.K., Batt C.A. (2000). MEAT AND POULTRY | Spoilage of Cooked Meats and Meat Products. Encyclopedia of Food Microbiology.

[39] Stellato G., La Storia A., de Filippis F., Borriello G., Villani F., Ercolini D. (2016). Overlap of Spoilage-Associated Microbiota between Meat and the Meat Processing Environment in Small-Scale and Large-Scale Retail Distributions. Appl. Environ. Microbiol..

[40] in’t Veld J.H.H. (1996). Microbial and biochemical spoilage of foods: An overview. Int. J. Food Microbiol..

[41] Mann E., Wetzels S.U., Pinior B., Metzler-Zebeli B.U., Wagner M., Schmitz-Esser S. (2016). Psychrophile spoilers dominate the bacterial microbiome in musculature samples of slaughter pigs. Meat Sci..

[42] Koutsoumanis K., Geornaras I., Sofos J. (2005). Microbiology of Land Muscle Foods. Handbook of Food Science, Technology, and Engineering, Taylor & Francis.

[43] Wickramasinghe N.N., Ravensdale J., Coorey R., Chandry S.P., Dykes G.A. (2019). The Predominance of Psychrotrophic Pseudomonads on Aerobically Stored Chilled Red Meat. Compr. Rev. Food Sci. Food Saf..

[44] Wickramasinghe N.N., Ravensdale J.T., Coorey R., Dykes G.A., Chandry P.S. (2019). In situ characterisation of biofilms formed by psychrotrophic meat spoilage pseudomonads. Biofouling.

[45] Ercolini D., Ferrocino I., Nasi A., Ndagijimana M., Vernocchi P., La Storia A., Laghi L., Mauriello G., Guerzoni M.E., Villani F. (2011). Monitoring of microbial metabolites and bacterial diversity in beef stored under different packaging conditions. Appl. Environ. Microbiol..

[46] Balamatsia C.C., Paleologos E.K., Kontominas M.G., Savvaidis I.N. (2006). Correlation between microbial flora, sensory changes and biogenic amines formation in fresh chicken meat stored aerobically or under modified atmosphere packaging at 4 degrees C: Possible role of biogenic amines as spoilage indicators. Antonie Van Leeuwenhoek.

[47] Hilgarth M., Lehner E.M., Behr J., Vogel R.F. (2019). Diversity and anaerobic growth of Pseudomonas spp. isolated from modified atmosphere packaged minced beef. J. Appl. Microbiol..

[48] Ercolini D., Casaburi A., Nasi A., Ferrocino I., Di Monaco R., Ferranti P., Mauriello G., Villani F. (2010). Different molecular types of Pseudomonas fragi have the same overall behaviour as meat spoilers. Int. J. Food Microbiol..

[49] Yushina Y.K., Bataeva D.S., Zaiko E.V., Machova A.A., Velebit B. (2019). Bacterial populations and volatile organic compounds associated with meat spoilage. IOP Conf. Ser. Earth Environ. Sci..

[50] Jääskeläinen E., Hultman J., Parshintsev J., Riekkola M.-L., Björkroth J. (2016). Development of spoilage bacterial community and volatile compounds in chilled beef under vacuum or high oxygen atmospheres. Int. J. Food Microbiol..

[51] Blixt Y., Borch E. (2002). Comparison of shelf life of vacuum-packed pork and beef. Meat Sci..

[52] Doulgeraki A.I., Paramithiotis S., Kagkli D.M., Nychas G.-J.E. (2010). Lactic acid bacteria population dynamics during minced beef storage under aerobic or modified atmosphere packaging conditions. Food Microbiol..

[53] Wambui J., Stephan R. (2019). Relevant Aspects of Clostridium estertheticum as a Specific Spoilage Organism of Vacuum-Packed Meat. Microorganisms.

[54] Broda D.M., Delacy K.M., Bell R., Braggins T.J., Cook R.L. (1996). Psychrotrophic Clostridium spp. associated with ‘blown pack’ spoilage of chilled vacuum-packed red meats and dog rolls in gas-impermeable plastic casings. Int. J. Food Microbiol..

[55] Djordjevic J., Boskovic M., Dokmanovic M., Lazic I.B., Ledina T., Suvajdzic B., Baltic M.Z. (2017). Vacuum and Modified Atmosphere Packaging Effect on Enterobacteriaceae Behaviour in Minced Meat. J. Food Process. Preserv..

[56] Moschonas G., Bolton D.J., Sheridan J.J., McDowell D.A. (2010). The effect of storage temperature and inoculum level on the time of onset of ‘blown pack’ spoilage. J. Appl. Microbiol..

[57] Liang C., Zhang D., Zheng X., Wen X., Yan T., Zhang Z., Hou C. (2021). Effects of Different Storage Temperatures on the Physicochemical Properties and Bacterial Community Structure of Fresh Lamb Meat. Food Sci. Anim. Resour..

[58] Kennedy J., Jackson V., Blair I.S., McDowell D.A., Cowan C., Bolton D.J. (2005). Food safety knowledge of consumers and the microbiological and temperature status of their refrigerators. J. Food Prot..

[59] Doulgeraki A.I., Ercolini D., Villani F., Nychas G.-J.E. (2012). Spoilage microbiota associated to the storage of raw meat in different conditions. Int. J. Food Microbiol..

[60] Bruckner S., Albrecht A., Petersen B., Kreyenschmidt J. (2012). Influence of cold chain interruptions on the shelf life of fresh pork and poultry. Int. J. Food Sci. Technol..

[61] Sørheim O., Nissen H., Nesbakken T. (1999). The storage life of beef and pork packaged in an atmosphere with low carbon monoxide and high carbon dioxide. Meat Sci..

[62] Holy A. (1988). The influence of extrinsic factors on the microbiological spoilage pattern of ground beef. Int. J. Food Microbiol..

[63] FSIS, U. (2002). FSIS Safety and Security Guidelines for the Transportation and Distribution of Meat, Poultry, and Egg Products. https://www.ams.usda.gov/sites/default/files/media/FSIS%20Safety%20and%20Security%20Guidelines%20for%20the%20Transportation%20and%20Distribution%20of%20Meat%2C%20Poultry%2C%20and%20Egg%20Products.pdf.

[64] (2004). Regulation (EC) No2004 OF The European Parliament and of the Council of 29 April 2004 Laying Down Specific Hygiene Rules for on the Hygiene of Foodstuffs.

[65] (2017). REGULATION C.O.M.S.I.N. 2017/1981 of 31 October 2017 Amending Annex III to Regulation (EC) No 853/2004 of the European Parliament and of the Council as Regards Temperature Conditions during Transport of Meat.

[66] Farber J.M. (1991). Microbiological Aspects of Modified-Atmosphere Packaging Technology—A Review 1. J. Food Prot..

[67] McMillin K.W. (2017). Advancements in meat packaging. Meat Sci..

[68] Arvanitoyannis I.S., Stratakos A.C. (2012). Application of Modified Atmosphere Packaging and Active/Smart Technologies to Red Meat and Poultry: A Review. Food Bioprocess Technol..

[69] McMillin K.W. (2008). Where is MAP Going? A review and future potential of modified atmosphere packaging for meat. Meat Sci..

[70] Wang T., Zhao L., Sun Y., Ren F., Chen S., Zhang H., Guo H. (2016). Changes in the microbiota of lamb packaged in a vacuum and in modified atmospheres during chilled storage analysed by high-throughput sequencing. Meat Sci..

[71] Luong N.-D.M., Coroller L., Zagorec M., Membré J.-M., Guillou S. (2020). Spoilage of Chilled Fresh Meat Products during Storage: A Quantitative Analysis of Literature Data. Microorganisms.

[72] Dhananjayan R., Han I.Y., Acton J.C., Dawson P.L. (2006). Growth depth effects of bacteria in ground turkey meat patties subjected to high carbon dioxide or high oxygen atmospheres. Poult. Sci..

[73] Dousset X., Jaffrès E., Zagorec M., Caballero B., Toldrá F., Finglas P.M. (2015). Spoilage: Bacterial Spoilage. Encyclopedia of Food and Health.

[74] Church N. (1994). Developments in modified-atmosphere packaging and related technologies. Trends Food Sci. Technol..

[75] Pennacchia C., Ercolini D., Villani F. (2011). Spoilage-related microbiota associated with chilled beef stored in air or vacuum pack. Food Microbiol..

[76] Fang Z., Zhao Y., Warner R.D., Johnson S.K. (2017). Active and intelligent packaging in meat industry. Trends Food Sci. Technol..

[77] Fernández-Pan I., Mendoza M., Maté J.I. (2013). Whey protein isolate edible films with essential oils incorporated to improve the microbial quality of poultry. J. Sci. Food Agric..

[78] Lorenzo J.M., Batlle R., Gómez M. (2014). Extension of the shelf-life of foal meat with two antioxidant active packaging systems. Food Sci. Technol..

[79] Stratakos A.C., Delgado-Pando G., Linton M., Patterson M.F., Koidis A. (2015). Synergism between high-pressure processing and active packaging against Listeria monocytogenes in ready-to-eat chicken breast. Innov. Food Sci. Emerg. Technol..

[80] Tornuk F., Hancer M., Sagdic O., Yetim H. (2015). LLDPE based food packaging incorporated with nanoclays grafted with bioactive compounds to extend shelf life of some meat products. LWT—Food Sci. Technol..

[81] Quesada J., Sendra E., Navarro C., Sayas-Barberá E. (2016). Antimicrobial Active Packaging including Chitosan Films with Thymus vulgaris L. Essent. Oil Readyto-Eat Meat. Foods.

[82] Tørngren M.A., Darré M., Gunvig A., Bardenshtein A. (2018). Case studies of packaging and processing solutions to improve meat quality and safety. Meat Sci..

[83] Jessen B., Lammert L. (2003). Biofilm and disinfection in meat processing plants. Int. Biodeterior. Biodegrad..

[84] Arnold J.W., Silvers S. (2000). Comparison of poultry processing equipment surfaces for susceptibility to bacterial attachment and biofilm formation. Poult. Sci..

[85] Giaouris E., Heir E., Hébraud M., Chorianopoulos N., Langsrud S., Møretrø T., Habimana O., Desvaux M., Renier S., Nychas G.-J. (2014). Attachment and biofilm formation by foodborne bacteria in meat processing environments: Causes, implications, role of bacterial interactions and control by alternative novel methods. Meat Sci..

[86] Reid R., Fanning S., Whyte P., Kerry J., Bolton D. (2017). Comparison of hot versus cold boning of beef carcasses on bacterial growth and the risk of blown pack spoilage. Meat Sci..

[87] Mills J., Donnison A., Brightwell G. (2014). Factors affecting microbial spoilage and shelf-life of chilled vacuum-packed lamb transported to distant markets: A review. Meat Sci..

[88] Stefanello A., Gasperini A.M., Copetti M.V. (2022). Ecophysiology of OTA-producing fungi and its relevance in cured meat products. Curr. Opin. Food Sci..

[89] Preetha S.S., Narayanan R. (2020). Factors Influencing the Development of Microbes in Food. Ash.

[90] Chmiel M., Roszko M., Hać-Szymańczuk E., Adamczak L., Florowski T., Pietrzak D., Cegiełka A., Bryła M. (2020). Time evolution of microbiological quality and content of volatile compounds in chicken fillets packed using various techniques and stored under different conditions. Poult. Sci..

[91] Sun X., Young J., Liu J.-H., Newman D. (2018). Prediction of pork loin quality using online computer vision system and artificial intelligence model. Meat Sci..

[92] Cleveland J., Montville T.J., Nes I.F., Chikindas M.L. (2001). Bacteriocins: Safe, natural antimicrobials for food preservation. Int. J. Food Microbiol..

[93] Pothakos V., Devlieghere F., Villani F., Björkroth J., Ercolini D. (2015). Lactic acid bacteria and their controversial role in fresh meat spoilage. Meat Sci..

[94] Argyri A.A., Mallouchos A., Panagou E.Z., Nychas G.-J.E. (2015). The dynamics of the HS/SPME-GC/MS as a tool to assess the spoilage of minced beef stored under different packaging and temperature conditions. Int. J. Food Microbiol..

[95] Papadopoulou O.S., Iliopoulos V., Mallouchos A., Panagou E.Z., Chorianopoulos N., Tassou C.C., Nychas G.-J.E. (2020). Spoilage Potential of Pseudomonas (P. fragi, P. putida) and LAB (Leuconostoc mesenteroides, Lactobacillus sakei) Strains and Their Volatilome Profile During Storage of Sterile Pork Meat Using GC/MS and Data Analytics. Foods.

[96] La Storia A., Ferrocino I., Torrieri E., Di Monaco R., Mauriello G., Villani F., Ercolini D. (2012). A combination of modified atmosphere and antimicrobial packaging to extend the shelf-life of beefsteaks stored at chill temperature. Int. J. Food Microbiol..

[97] Domínguez R., Pateiro M., Gagaoua M., Barba F.J., Zhang W., Lorenzo J.M. (2019). A Comprehensive Review on Lipid Oxidation in Meat and Meat Products. Antioxidants.

[98] Saraiva C., Oliveira I., Silva J.A., Martins C., Ventanas J., García C. (2015). Implementation of multivariate techniques for the selection of volatile compounds as indicators of sensory quality of raw beef. J. Food Sci. Technol..

[99] Nieminen T.T., Dalgaard P., Björkroth J. (2016). Volatile organic compounds and Photobacterium phosphoreum associated with spoilage of modified-atmosphere-packaged raw pork. Int. J. Food Microbiol..

[100] Zhou C., Zhan G., Pan D., Zhou G., Wang Y., He J., Cao J. (2022). Charactering the spoilage mechanism of “three sticks” of Jinhua ham. Food Sci. Hum. Wellness.

[101] Liu Z.-Y., Zhou D.-Y., Li A., Zhao M.-T., Hu Y.-Y., Li D.-Y., Xie H.-K., Zhao Q., Hu X.-P., Zhang J.-H. (2020). Fereidoon Shahidi Effects of temperature and heating time on the formation of aldehydes during the frying process of clam assessed by an HPLC-MS/MS method. Food Chem..

[102] Mayr D., Margesin R., Klingsbichel E., Hartungen E., Jenewein D., Schinner F., Märk T.D. (2003). Rapid detection of meat spoilage by measuring volatile organic compounds by using proton transfer reaction mass spectrometry. Appl. Environ. Microbiol..

[103] Rogers H.B., Brooks J.C., Martin J.N., Tittor A., Miller M.F., Brashears M.M. (2014). The impact of packaging system and temperature abuse on the shelf life characteristics of ground beef. Meat Sci..

[104] Cayuela J., Gil M., Ban S., Garrido M. (2004). Effect of vacuum and modified atmosphere packaging on the quality of pork loin. Eur. Food Res. Technol..

[105] John L., Cornforth D., Carpenter C.E., Sorheim O., Pettee B.C., Whittier D.R. (2005). Color and thiobarbituric acid values of cooked top sirloin steaks packaged in modified atmospheres of 80% oxygen, or 0.4% carbon monoxide, or vacuum. Meat Sci..

[106] Ioannidis A.-G., Walgraeve C., Vanderroost M., van Langenhove H., Devlieghere F., de Meulenaer B. (2018). Non-Destructive Measurement of Volatile Organic Compounds in Modified Atmosphere Packaged Poultry Using SPME-SIFT-MS in Tandem with Headspace TD-GC-MS. Food Anal. Methods.

[107] Sirocchi V., Caprioli G., Cecchini C., Coman M.M., Cresci A., Maggi F., Papa F., Ricciutelli M., Vittori S., Sagratini G. (2013). Biogenic amines as freshness index of meat wrapped in a new active packaging system formulated with essential oils of Rosmarinus officinalis. Int. J. Food Sci. Nutr..

[108] Sun Y., Fu M., Li Z., Peng X. (2018). Evaluation of Freshness in Determination of Volatile Organic Compounds Released from Pork by HS-SPME-GC-MS. Food Anal. Methods.

[109] Casaburi A., Nasi A., Ferrocino I., Di Monaco R., Mauriello G., Villani F., Ercolini D. (2011). Spoilage-related activity of Carnobacterium maltaromaticum strains in air-stored and vacuum-packed meat. Appl. Environ. Microbiol..

[110] Borch E., Agerhem H. (1992). Chemical, microbial and sensory changes during the anaerobic cold storage of beef inoculated with a homofermentative Lactobacillus sp. or a Leuconostoc sp. Int. J. Food Microbiol..

[111] Mikš-Krajnik M., Yoon Y.-J., Ukuku D.O., Yuk H.-G. (2016). Identification and Quantification of Volatile Chemical Spoilage Indexes Associated with Bacterial Growth Dynamics in Aerobically Stored Chicken. J. Food Sci..

[112] Sedgwick P. (2012). Pearson’s correlation coefficient. BMJ.

[113] Stutz H., Silverman G.J., Angelini P., Levin R.E. (1991). Bacteria and Volatile Compounds Associated with Ground Beef Spoilage. J. Food Sci..

[114] Nychas G.J., Arkoudelos J.S. (1990). Microbiological and physicochemical changes in minced meats under carbon dioxide, nitrogen or air at 3 °C. Int. J. Food Sci. Technol..

[115] Mansur A.R., Seo D.-H., Song E.-J., Song N.-E., Hwang S.H., Yoo M., Nam T.G. (2019). Identifying potential spoilage markers in beef stored in chilled air or vacuum packaging by HS-SPME-GC-TOF/MS coupled with multivariate analysis. LWT—Food Sci. Technol..

[116] Nychas G.-J.E., Skandamis P.N., Tassou C.C., Koutsoumanis K.P. (2008). Meat spoilage during distribution. Meat Sci..

[117] Ercolini D., Russo F., Nasi A., Ferranti P., Villani F. (2009). Mesophilic and psychrotrophic bacteria from meat and their spoilage potential in vitro and in beef. Appl. Environ. Microbiol..

[118] Nurjuliana M., Man Y.B.C., Hashim D.M., Mohamed A.K.S. (2011). Rapid identification of pork for halal authentication using the electronic nose and gas chromatography mass spectrometer with headspace analyzer. Meat Sci..

[119] Chen L., Mardiansyah S.T., Kuuliala L., Somrani Achouri M., Walgraeve C., Demeestere K., Devlieghere F. (2022). Identification of Volatile Spoilage Indicators for Pork Packaged under Modified Atmospheres. Dublin. https://biblio.ugent.be/publication/01GJ59ACV4XBS7A3MACD9QS05M.

[120] Mansur A.R., Song E.-J., Cho Y.-S., Nam Y.-D., Choi Y.-S., Kim D.-O., Seo D.-H., Nam T.G. (2019). Comparative evaluation of spoilage-related bacterial diversity and metabolite profiles in chilled beef stored under air and vacuum packaging. Food Microbiol..

[121] Ghasemi-Varnamkhasti M., Apetrei C., Lozano J., Anyogu A. (2018). Potential use of electronic noses, electronic tongues and biosensors as multisensor systems for spoilage examination in foods. Trends Food Sci. Technol..

[122] Zareian M., Böhner N., Loos H.M., Silcock P., Bremer P., Beauchamp J. (2018). Evaluation of volatile organic compound release in modified atmosphere-packaged minced raw pork in relation to shelf-life. Food Packag. Shelf Life.

[123] Mikš-Krajnik M., Yoon Y.-J., Yuk H.-G. (2015). Detection of volatile organic compounds as markers of chicken breast spoilage using HS-SPME-GC/MS-FASST. Food Sci. Biotechnol..

[124] Insausti K., Beriain M.J., Gorraiz C., Purroy A. (2002). Volatile Compounds of Raw Beef from 5 Local Spanish Cattle Breeds Stored Under Modified Atmosphere. J Food Sci..

[125] Hughes M.N., Centelles M.N., Moore K.P. (2009). Making and working with hydrogen sulfide: The chemistry and generation of hydrogen sulfide in vitro and its measurement in vivo: A review. Free Radic. Biol. Med..

[126] Eilamo M., Kinnunen A., Latva-Kala K., Ahvenainen R. (1998). Effects of packaging and storage conditions on volatile compounds in gas-packed poultry meat. Food Addit. Contam..

[127] Comi G. (2017). Spoilage of Meat and Fish.

[128] Dainty R.H. (1996). Chemical/biochemical detection of spoilage. Int. J. Food Microbiol..

[129] Koskela J., Sarfraz J., Ihalainen P., Määttänen A., Pulkkinen P., Tenhu H., Nieminen T., Kilpelä A., Peltonen J. (2015). Monitoring the quality of raw poultry by detecting hydrogen sulfide with printed sensors. Sens. Actuators B Chem..

[130] Toldra F. (1998). Proteolysis and lipolysis in flavour development of dry-cured meat products. Meat Sci..

[131] Martín A., Benito M.J., Aranda E., Ruiz-Moyano S., Córdoba J.J., Córdoba M.G. (2010). Characterization by volatile compounds of microbial deep spoilage in Iberian dry-cured ham. J. Food Sci..

[132] Kakouri A., Nychas G.-J.E. (1994). Storage of poultry meat under modified atmospheres or vacuum packs: Possible role of microbial metabolites as indicator of spoilage. J. Appl. Bacteriol..

[133] Rosa M.D., Galanakis C.M. (2019). Packaging Sustainability in the Meat Industry. Sustainable Meat Production and Processing.

[134] Hernandez M.S., Woerner D.R., Brooks J.C., Wheeler T.L., Legako J.F. (2022). Influence of Aging Temperature and Duration on Spoilage Organism Growth, Proteolytic Activity, and Related Chemical Changes in Vacuum-Packaged Beef Longissimus. Meat and Muscle Biology.

[135] Ayseli M.T., Filik G., Selli S. (2014). Evaluation of volatile compounds in chicken breast meat using simultaneous distillation and extraction with odour activity value. J. Food Nutr. Res..

[136] Cortez-Vega W.R., Pizato S., Prentice C. (2012). Quality of raw chicken breast stored at 5 °C and packaged under different modified atmospheres. J Food Saf..

[137] Nam K., Ahn D. (2003). Combination of aerobic and vacuum packaging to control lipid oxidation and off-odor volatiles of irradiated raw turkey breast. Meat Sci..

[138] Li M., Tian L., Zhao G., Zhang Q., Gao X., Huang X., Sun L. (2014). Formation of biogenic amines and growth of spoilage-related microorganisms in pork stored under different packaging conditions applying PCA. Meat Sci..

[139] Sawaya W.N., Elnawawy A.S., Al-Zenki S., Al-Otaibi J., Al-Omirah H., Al-Amiri H. (1995). Storage Stability of Chicken as Affected by Map and Lactic Acid Treatment. J Food Sci..

[140] Nychas G.J., Dillon V.M., Board R.G. (1988). Glucose, the Key Substrate in the Microbiological Changes Occurring in Meat and Certain Meat Products. Biotechnol. Appl. Biochem..

[141] Odeyemi O.A., Alegbeleye O.O., Strateva M., Stratev D. (2020). Understanding spoilage microbial community and spoilage mechanisms in foods of animal origin. Compr. Rev. Food Sci. Food Saf..

[142] Rokka M., Eerola S., Smolander M., Alakomi H.-L., Ahvenainen R. (2004). Monitoring of the quality of modified atmosphere packaged broiler chicken cuts stored in different temperature conditions. Food Control.

[143] Hutarova Z., Vecerek V., Steinhauserova I., Marsalek P., Borilova G., Forejtek P. (2013). The effect of treating method of pithed pheasant on the content of biogenic amines in the meat during the course of storage. Poult. Sci..

[144] Jairath G., Singh P.K., Dabur R.S., Rani M., Chaudhari M. (2015). Biogenic amines in meat and meat products and its public health significance: A review. J. Food Sci. Technol..

[145] Lázaro C.A., Junior C.A.C., Canto A.C., Monteiro M.L.G., Franco R.M. (2015). Biogenic amines as bacterial quality indicators in different poultry meat species. LWT—Food Sci. Technol..

[146] Silva C.M., Glória M.A. (2002). Bioactive amines in chicken breast and thigh after slaughter and during storage at 4±1 °C and in chicken-based meat products. Food Chem..

[147] Schirone M., Esposito L., D’onofrio F., Visciano P., Martuscelli M., Mastrocola D., Paparella A. (2022). Biogenic Amines in Meat and Meat Products: A Review of the Science and Future Perspectives. Foods.

[148] Ruiz-Capillas C., Moral A. (2004). Free amino acids and biogenic amines in red and white muscle of tuna stored in controlled atmospheres. Amino Acids.

[149] Naila A., Flint S., Fletcher G., Bremer P., Meerdink G. (2010). Control of biogenic amines in food--existing and emerging approaches. J. Food Sci..

[150] Ahmad W., Mohammed G.I., Al-Eryani D.A., Saigl Z.M., Alyoubi A.O., Alwael H., Bashammakh A.S., O’sullivan C.K., El-Shahawi M.S. (2019). Biogenic Amines Formation Mechanism and Determination Strategies: Future Challenges and Limitations. Crit. Rev. Anal. Chem..

[151] Latorre-Moratalla M.L., Bover-Cid S., Bosch-Fusté J., Veciana-Nogués M.T., Vidal-Carou M.C. (2014). Amino acid availability as an influential factor on the biogenic amine formation in dry fermented sausages. Food Control.

[152] Krizek A.R., Smith J.S., Phebus R.K. (1995). Biogenic Amine Formation in Fresh Vacuum-Packaged Beef Stored at −2 °C and 2 °C for 100 Days. J. Food Prot..

[153] Geornaras I., Dykes G.A., von Holy A. (1995). Biogenic amine formation by poultry-associated spoilage and pathogenic bacteria. Lett. Appl. Microbiol..

[154] Galgano F., Favati F., Bonadio M., Lorusso V., Romano P. (2009). Role of biogenic amines as index of freshness in beef meat packed with different biopolymeric materials. Food Res. Int..

[155] Moreira A.P.S., Giombelli A., Labanca R.A., Nelson D.L., Glória M.B.A. (2008). Effect of aging on bioactive amines, microbial flora, physico-chemical characteristics, and tenderness of broiler breast meat. Poult. Sci..

[156] Rosinská D., Lehotay J. (2014). INFLUENCE OF TEMPERATURE ON PRODUCTION OF BIOGENIC AMINES IN PORK, BEEF, AND POULTRY AND THEIR HPLC DETERMINATION AFTER POSTCOLUMN DERIVATIZATION. J. Liq. Chromatogr. Relat. Technol..

[157] Gardini F., Özogul Y., Suzzi G., Tabanelli G., Özogul F. (2016). Technological Factors Affecting Biogenic Amine Content in Foods: A Review. Front. Microbiol..

[158] Durlu-Özkaya F., Ayhan K., Vural N. (2001). Biogenic amines produced by Enterobacteriaceae isolated from meat products. Meat Sci..

[159] Bover-Cid S., Holzapfel W.H. (1999). Improved screening procedure for biogenic amine production by lactic acid bacteria. Int. J. Food Microbiol..

[160] Min J.S., Lee S.O., Jang A., Jo C., Lee M. (2007). Control of microorganisms and reduction of biogenic amines in chicken breast and thigh by irradiation and organic acids. Poult. Sci..

[161] Chmiel M., Roszko M., Hać-Szymańczuk E., Cegiełka A., Adamczak L., Florowski T., Pietrzak D., Bryła M., Świder O. (2022). Changes in the microbiological quality and content of biogenic amines in chicken fillets packed using various techniques and stored under different conditions. Food Microbiol..

[162] Vinci G., Antonelli M. (2002). Biogenic amines: Quality index of freshness in red and white meat. Food Control.

[163] Baston O., Barna O., Vasile A. (2010). Microbiota and biogenic amines variation of chicken meat. Comparison between white and red meat. Ann. Food Sci. Technol..

[164] Alessandroni L., Caprioli G., Faiella F., Fiorini D., Galli R., Huang X., Marinelli G., Nzekoue F., Ricciutelli M., Scortichini S. (2022). A shelf-life study for the evaluation of a new biopackaging to preserve the quality of organic chicken meat. Food Chem..

[165] Dudnyk I., Janeček E.-R., Vaucher-Joset J., Stellacci F. (2018). Edible sensors for meat and seafood freshness. Sens. Actuators B Chem..

[166] Cheng W., Sun D.-W., Cheng J.-H. (2016). Pork biogenic amine index (BAI) determination based on chemometric analysis of hyperspectral imaging data. LWT—Food Sci. Technol..

[167] Hernández-Jover T., Izquierdo-Pulido M., Veciana-Nogués M.T., Vidal-Carou M.C. (1996). Biogenic Amine Sources in Cooked Cured Shoulder Pork. J. Agric. Food Chem..

[168] Mayr D., Margesin R., Schinner F., Märk T. (2003). Detection of the spoiling of meat using PTR–MS. Int. J. Mass Spectrom..

[169] Wang Y., Zhang W., Fu L. (2017). Food Spoilage Microorganisms: Ecology and Control.

[170] Randell K., Ahvenainen R., Latva-Kala K., Hurme E., Mattila-Sandholm T., Hyvönen L. (1995). Modified Atmosphere-packed Marinated Chicken Breast and Rainbow Trout Quality as Affected by Package Leakage. J. Food Sci..

[171] Kuswandi B., Nurfawaidi A. (2017). On-package dual sensors label based on pH indicators for real-time monitoring of beef freshness. Food Control.

[172] Blacha I., Krischek C., Klein G. (2014). Influence of modified atmosphere packaging on meat quality parameters of turkey breast muscles. J. Food Prot..

[173] Ezati P., Tajik H., Moradi M. (2019). Fabrication and characterization of alizarin colorimetric indicator based on cellulose-chitosan to monitor the freshness of minced beef. Sens. Actuators B Chem..

[174] Fraqueza M.J., Alfaia C.M., Barreto A.S. (2012). Biogenic amine formation in turkey meat under modified atmosphere packaging with extended shelf life: Index of freshness. Poult. Sci..

[175] Qiao L., Tang X., Dong J. (2017). A feasibility quantification study of total volatile basic nitrogen (TVB-N) content in duck meat for freshness evaluation. Food Chem..

[176] Bekhit A.E.-D.A., Holman B.W., Giteru S.G., Hopkins D.L. (2021). Total volatile basic nitrogen (TVB-N) and its role in meat spoilage: A review. Trends Food Sci. Technol..

[177] Balamatsia C.C., Patsias A., Kontominas M.G., Savvaidis I.N. (2007). Possible role of volatile amines as quality-indicating metabolites in modified atmosphere-packaged chicken fillets: Correlation with microbiological and sensory attributes. Food Chem..

[178] Wójcik W., Łukasiewicz M., Puppel K. (2021). Biogenic amines: Formation, action and toxicity—A review. J. Sci. Food Agric..

[179] Brink B.T., Damink C., Joosten H., Huis in ’t Veld J.H.J. (1990). Occurrence and formation of biologically active amines in foods. Int. J. Food Microbiol..

[180] Stadnik J., Dolatowski Z.J. (2010). Biogenic amines in meat and fermented meat products. Acta Sci.Pol. Technol. Aliment..

[181] Magnaghi L.R., Alberti G., Capone F., Zanoni C., Mannucci B., Quadrelli P., Biesuz R. (2020). Development of a Dye-Based Device to Assess the Poultry Meat Spoilage. Part II Array Act. J. Agric. Food Chem..

[182] Hernández-Jover T., Izquierdo-Pulido M., Veciana-Nogués M.T., Mariné-Font A., Vidal-Carou M.C. (1997). Effect of Starter Cultures on Biogenic Amine Formation during Fermented Sausage Production. J. Food Prot..

